# Recent applications of ninhydrin in multicomponent reactions

**DOI:** 10.1039/d0ra02930k

**Published:** 2020-05-18

**Authors:** Suven Das

**Affiliations:** Department of Chemistry, Rishi Bankim Chandra College for Women Naihati, 24-Parganas (N) 743165 India suvenchem@yahoo.co.in

## Abstract

Ninhydrin (1,2,3-indanetrione hydrate) has a remarkable breadth in different fields, including organic chemistry, biochemistry, analytical chemistry and the forensic sciences. For the past several years, it has been considered an important building block in organic synthesis. Therefore, there is increasing interest in ninhydrin-based multicomponent reactions to rapidly build versatile scaffolds. Most of the works described here are simple reactions with readily available starting materials that result in complex molecular architectures. Some of the synthesized compounds exhibit interesting biological activities and constitute a new hope for anticancer agents. The present review aims to highlight the multicomponent reactions of ninhydrin towards diverse organic molecules during the period from 2014 to 2019.

## Abstract 2

This article aims to review recent multicomponent reactions of ninhydrin towards diverse organic scaffolds, such as indeno-fused heterocycles, spiro-indeno heterocycles, quinoxalines, propellanes, cage-like compounds, and dispiro heterocycles.

## Graphical abstract

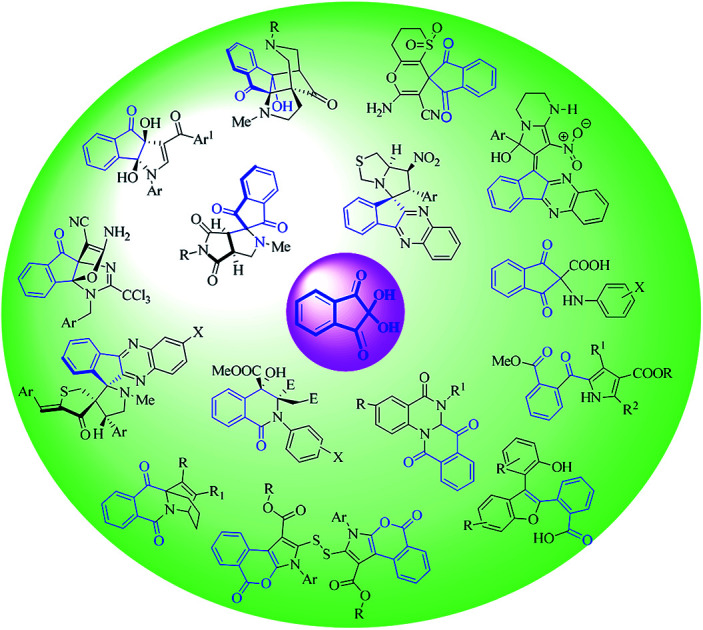

## 1. Introduction

**Fig. 1:**
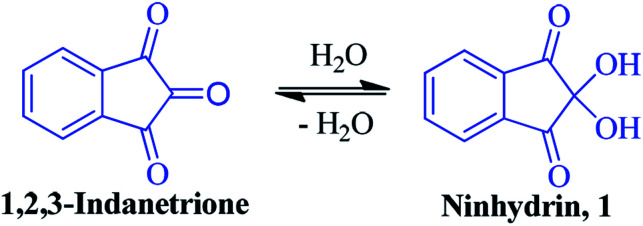
Structure of ninhydrin.

The compound ninhydrin 1 was first reported in the literature by English chemist S. Ruhemann more than a century ago.^1^ It is a stable, hydrated product of 1,2,3-indanetrione, where two hydroxyl groups at the C-2 position are flanked by two carbonyl groups (Fig. 1). Upon dehydration, the central carbonyl of the resulting indanetrione becomes the most reactive centre towards nucleophiles.^2,3^ In fact, ninhydrin is a strong electrophile that reacts with nucleophiles such as ammonia, amines, enamines, ureas, amides and anilines.^2–6^ Primary amines and α-amino acids react readily with ninhydrin at the central carbon to produce a highly coloured, condensation product known as Ruhemann's purple. Besides nitrogen-based nucleophiles, its C-2 position is reactive towards various carbon-, oxygen- and sulphur-based nucleophiles, resulting in C–C, C–O and C–S bonds, respectively.^7–14^ Due to its unique chemical structure and capability to form a dehydrated triketone analogue, it has the potential to act as a building block in diverse organic synthesis strategies.^15–21^

Furthermore, ninhydrin has special applications in the field of fluorescence. It is most widely applied as a reagent for the determination of latent fingerprints in forensic science.^22–24^ The fluorogenic ninhydrin reaction was reported for the assay of primary amines.^25^ It has been used as a potential substance for the micromolar determination of human serum albumin based on chemiluminescence.^26^ Recently, we employed monoarylated ninhydrin-adducts to develop a new fluorophore system.^27,28^ Some indanone-based fluorophores were also explored to act as a receptor for specific metal ions.^29,30^

**Fig. 2:**
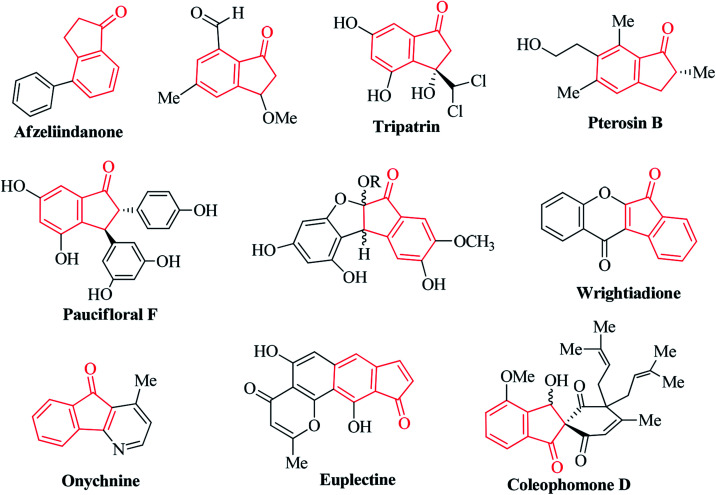
Some examples of natural products containing the indanone motif.

**Fig. 3:**
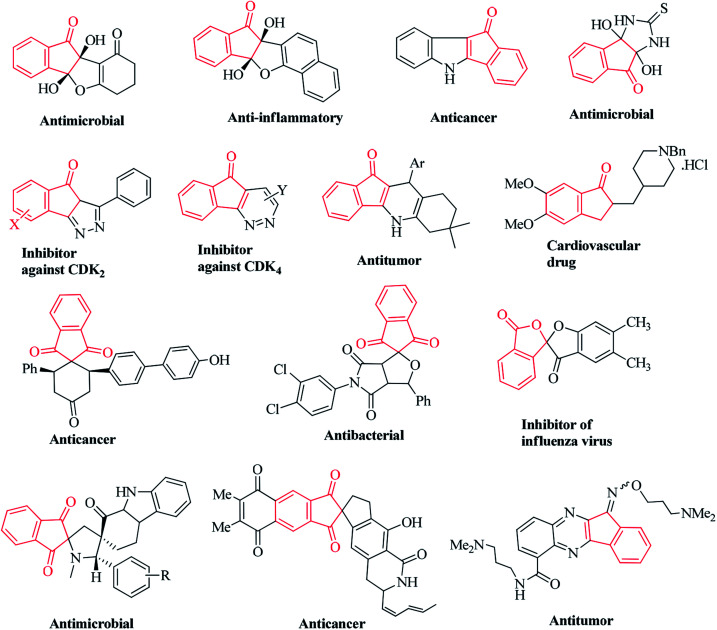
Representative examples of bioactive compounds containing the indanone skeleton.

Ninhydrin is basically an indanone class compound, and indanone core structures have been found in numerous natural products (Fig. 2).^31–40^ Indanone derivatives have demonstrated a broad spectrum of biological properties (Fig. 3). Some of the derivatives are well-known for their antimicrobial, anti-inflammatory, antagonistic, anti-allergy, anti-tumor, anti-cancer, and free radical scavenging activities.^41–49^ Spirocyclic indanones are prevalent in nature and possess pronounced pharmacological profiles.^50–56^ Moreover, heterocycle-fused indanone scaffolds are well recognized for their significant applications in medicinal chemistry.^57–62^ Several indenoquinoxaline scaffolds have been reported to function as potential anticancer agents.^61,62^

On the other hand, the multicomponent reaction (MCR) is a powerful synthetic tool for designing and developing a new route towards novel and complex molecular structures.^63–65^ In MCRs, three or more starting materials react in a single step to form a product that has substantial portions of all reactants. This strategy provides a high-throughput generation of combinatorial compound libraries in drug discovery research.^66–68^ Importantly, MCRs comply with the principles of green chemistry by saving reagents, solvents and time, while including the high atom-economy and selectivity of a reaction. In recent years, ninhydrin has become an unparalleled tricarbonyl compound participating in many MCRs to afford diverse structural scaffolds. It is worth mentioning that vicinal tricarbonyl compounds are rich sources of heterocyclic scaffolds.^69–72^ A review article was previously published on ninhydrin by Ziarani *et al.* regarding the synthesis of heterocyclic compounds until 2013.^73^ This review aims to highlight important MCRs of ninhydrin reported from 2014 to 2019.

## 2. Synthesis of indeno-fused heterocycles

**Scheme 1:**
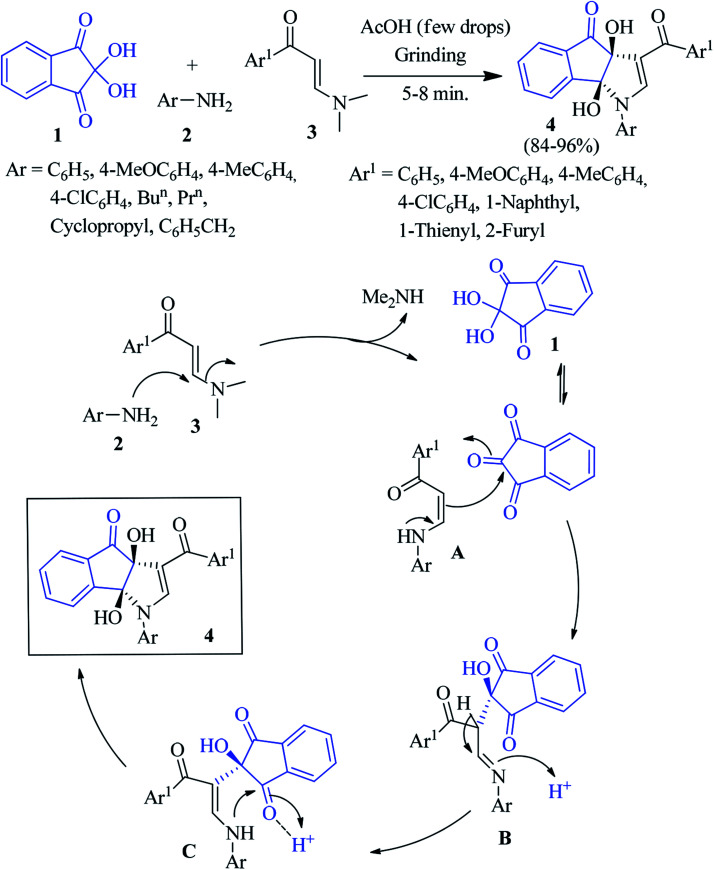
Synthesis of dihydroindeno[1,2-*b*]pyrrole derivative 4.

In 2014, Perumal and co-workers reported that the reaction of ninhydrin 1 with aniline 2 and (*E*)-3-(dimethylamino)-1-arylprop-2-en-1-one 3 in the presence of a catalytic amount of AcOH led to the formation of dihydroindeno[1,2-*b*]pyrrole 4 in excellent yield (Scheme 1).^74^ The facile, solvent-free, three component domino reaction afforded the regio- and stereoselective synthesis of the highly functionalized products at room temperature within 5–8 minutes under grinding condition. This green approach allowed the formation of two C–C and one C–N bonds in a single synthetic operation at ambient temperature. The reaction was initiated *via* Michael addition of aniline 2 to 3, followed by the elimination of Me_2_NH to yield intermediate A, which added the central carbonyl of ninhydrin chemoselectively to produce intermediate B. Then B underwent isomerisation to produce the enaminone pendant indanone intermediate C. Finally, annulation afforded the desired product 4.

**Scheme 2:**
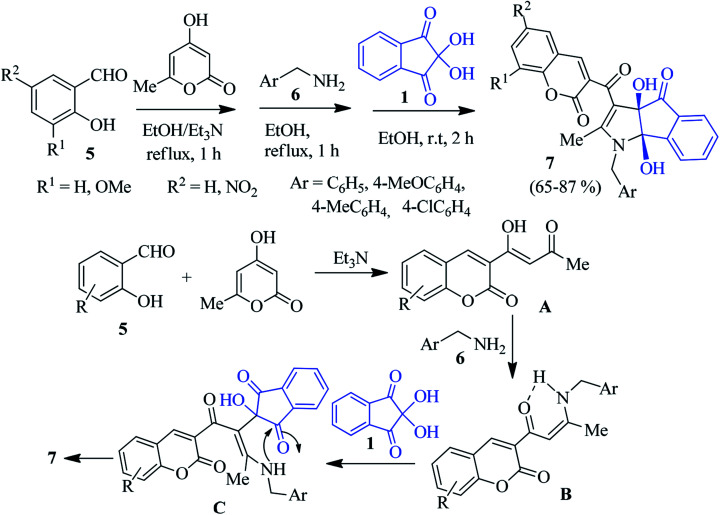
Synthesis of coumarin-appended indenopyrrole derivative 7.

Alizadeh described an excellent study *via* a one-pot four-component reaction involving salicylaldehyde 5, 4-hydroxy-6-methyl-2*H*-pyran-2-one, benzylamine 6 and ninhydrin 1 to access the potentially bioactive coumarin-appended indenopyrrole derivative 7 (Scheme 2).^75^ Different salicylaldehydes and benzylamines bearing electron donating and withdrawing substituents were reacted smoothly to deliver the products in good yields. In the presence of an Et_3_N catalyst, the Knoevenagel condensation between salicylaldehyde 5 and 4-hydroxy-6-methyl-2*H*-pyran-2-one leads to intermediate A. After condensation with benzylamine 6, this product forms the enamine intermediate B. Nucleophilic addition of B with ninhydrin 1 provides intermediate C. Finally, cyclization furnishes product 7 (Scheme 2).

**Scheme 3:**
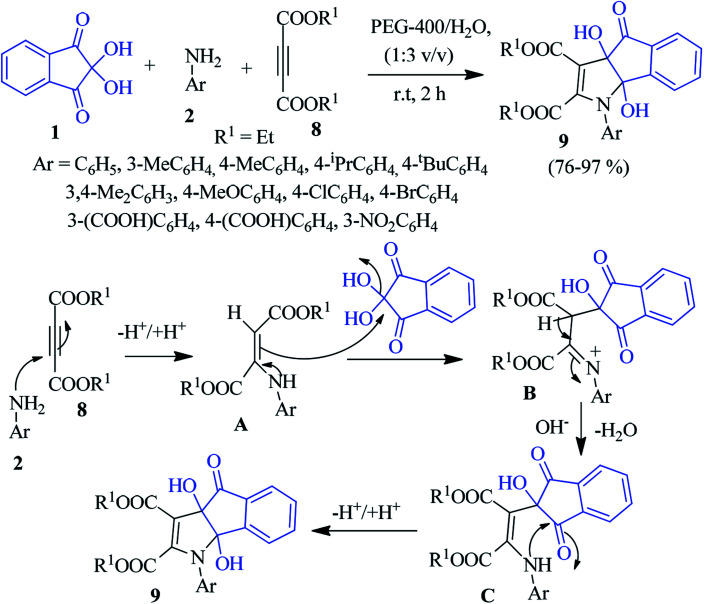
Green synthesis of dihydroindenopyrrole 9.

A new class of ninhydrin-based organic molecular probes, namely, dihydroindenopyrrole 9 was synthesized by Mukhopadhyay *et al.* In non-toxic polyethylene glycol-water (PEG 400-water), the reaction between ninhydrin 1, aniline 2 and dialkyl acetylenedicarboxylate (DAAD) 8 proceeded smoothly to achieve the novel product 9.^76^ A plausible mechanism is shown in Scheme 3, where amine 2 reacts with diester 8 to produce the enaminediester intermediate A. Then, intermediate A acts as a nucleophile to attack the central carbonyl of 1 to obtain intermediate B, which generates intermediate C upon dehydration. Next, the intramolecular cyclization results in the desired heterocyclic product 9. Interestingly, the synthesized compounds act as a sensor for the selective detection of the Al^3+^ ion through an off–on fluorescence response.

**Scheme 4:**
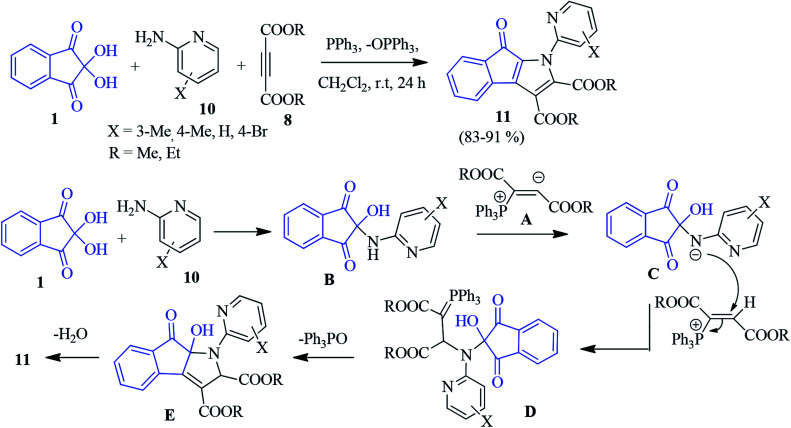
Synthesis of polysubstituted indenopyrroles 11.

A PPh_3_-promoted synthesis of the polysubstituted indenopyrrole 11 was accomplished through a three-component intramolecular Wittig reaction.^77^ The construction of the heterocylic skeleton was achieved by an annulation strategy involving ninhydrin 1, 2-aminopyridine 11 and DAAD 8 under acid or base-free conditions. Initially, zwitterion A was produced from the reaction of triphenylphosphene and acetylenic ester 8. Then, the zwitterion was protonated by the ninhydrin adduct B to generate the positively charged phosphonium ion. This ion was subsequently attacked by the intermediate C, leading to phosphorane D. An intramolecular Wittig reaction followed by dehydration furnished compound 11 (Scheme 4).

**Scheme 5:**
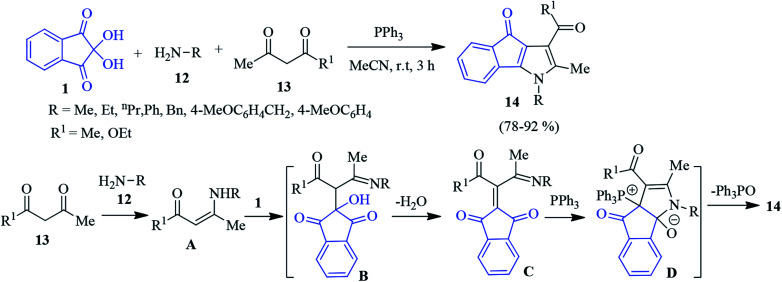
Synthesis of substituted indenopyrroles 14.

A similar version of the one-pot three component reaction was carried out employing 1, aliphatic amine 12 and 1,3-dicarbonyl compound 13 to access the indenopyrrole derivative 14.^78^ A plausible mechanism is depicted in Scheme 5. Enaminone A (produced from the reaction of 1,3-dicarbonyl 13 and amine 12) attacks ninhydrin 1 to generate intermediate B. Adduct B after dehydration offers intermediate C, which further reacts with triphenyl phosphene to produce zwitterion D. Finally, the elimination of triphenyl phosphene oxide affords target compound 14.

**Scheme 6:**
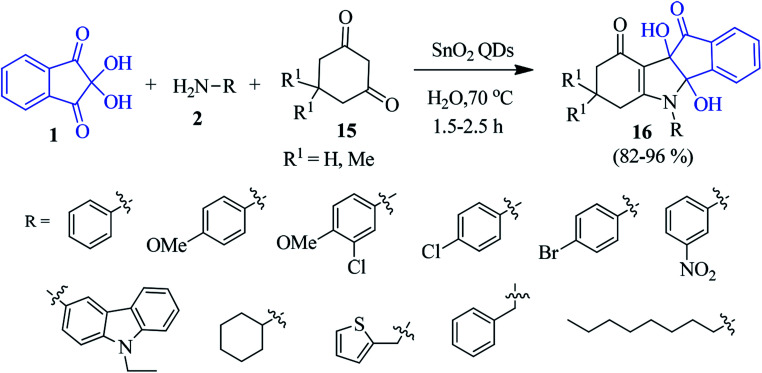
Green synthesis of indeno[1,2-*b*]indole derivatives 16.

Tin dioxide quantum dot (SnO_2_ QD) has been introduced as an efficient catalyst for preparing the indeno[1,2-*b*]indole derivative 16 by a three-component reaction of ninhydrin 1, amine 2 and cyclic 1,3-dicarbonyl compound 15 in an aqueous medium (Scheme 6).^79^ A variety of functional groups were compatible under the sustainable condition where the catalyst was reused for seven cycles with almost unaltered catalytic activity.

**Scheme 7:**
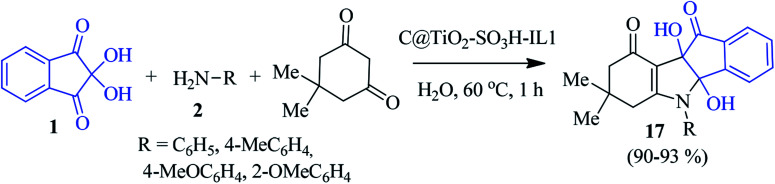
Green synthesis of indeno[1,2-*b*]indolone derivative 17.

Later, a novel ionic liquid coated sulfonated carbon@titania composite (C@TiO_2_-SO_3_H-IL1) was prepared and applied by the Paul group to access the indeno[1,2-*b*]indolone derivative 17. They performed the synthesis with ninhydrin 1, aniline 2 and dimedone in an aqueous medium in the presence of the aforesaid catalyst (Scheme 7).^80^ The newly designed catalyst showed remarkable activity and stability in water, resulting in an excellent yield of the product. The environmentally benign method allows for easy recovery of the catalyst for up to five cycles without a considerable loss of activity.

**Scheme 8:**
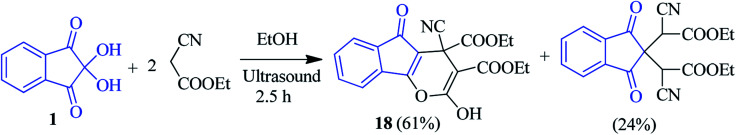
Synthesis of indenopyran derivative 18.

Kapoor *et al.* reported in their study that the reaction of ninhydrin 1 with 2 equivalents of ethyl cyanoacetate resulted in the formation of indenopyran derivative 18 (Scheme 8).^81^ The highly reactive C-2 of ninhydrin has been exploited to condense with an active methylene compound, resulting in 18 as the major product. Importantly, the reaction was carried out under an ultrasound condition without using any catalyst.

**Scheme 9:**
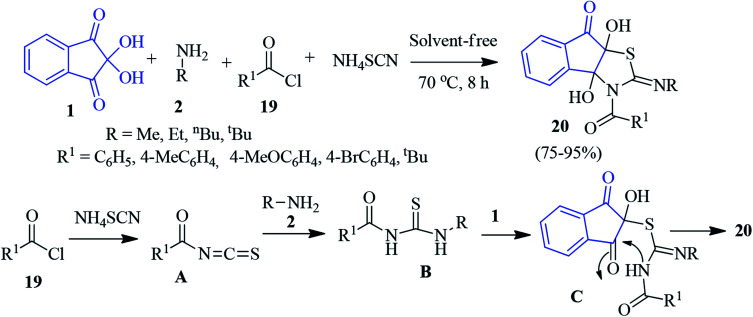
Solvent-free synthesis of indenothiazole derivative 20.

A facile one-pot four-component reaction of ninhydrin 1, primary amine 2, acid chloride 19 and ammonium thiocyanate was disclosed by Moradi to accomplish indenothiazole derivative 20 under solvent-free conditions (Scheme 9).^82^ Initially, the reaction of ammonium thiocyanate and acid chloride 19 led to the formation of alkanoyl isothiocyanate A. Then, intermediate A suffered a nucleophilic attack by amine 2 to form thiourea B. Subsequently, it attacked the central carbonyl of ninhydrin to produce intermediate C, which furnished compound 20 after annulation.

## 3. Synthesis of spiro-indanone-bearing N-heterocycles (*via* azomethine ylide)

Ninhydrin has been successfully employed for the construction of the spiro indanone framework anchored with various N-heterocyclic scaffolds. It should be mentioned that ninhydrin-derived azomethine ylides have been exploited to react with different dipolarophiles through the [3 + 2] cycloaddition towards the formation of various heterocyclic scaffolds.

**Scheme 10:**
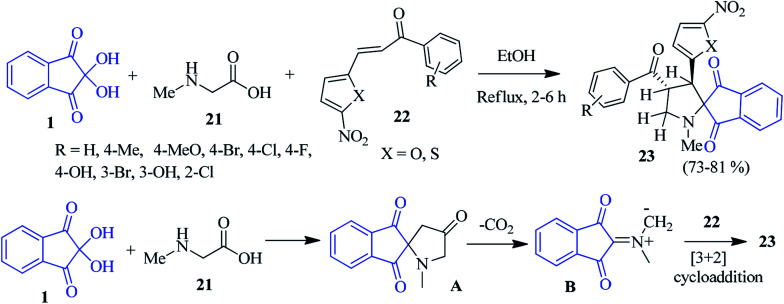
Regioselective synthesis of spiroindeno-pyrrolidine 23.

Kalluraya's group demonstrated a facile method for the synthesis of nitrofuran-bearing spiroindeno-pyrrolidines 23*via* a one-pot three component reaction of sarcosine 21, ninhydrin 1 and chalcone 22.^83^ The reaction proceeded with high regioselectivity in moderate to excellent yields in refluxing EtOH. Mechanistically, it is conceivable that sarcosine 21 and ninhydrin 1 reacted readily to form intermediate A. After decarboxylation, the *in situ* generated azomethine ylide B underwent a [3 + 2] cycloaddition with the dipolarophile 22, resulting in only one regioisomer as the cycloadduct 23 (Scheme 10). Inspired by the work described above, they developed a microwave-assisted solvent-free synthesis of nitrothiophene containing spiroindeno-pyrrolidines involving 1, 21 and nitrothiophene bearing chalcone 22^84^ after annulation.

**Scheme 11:**
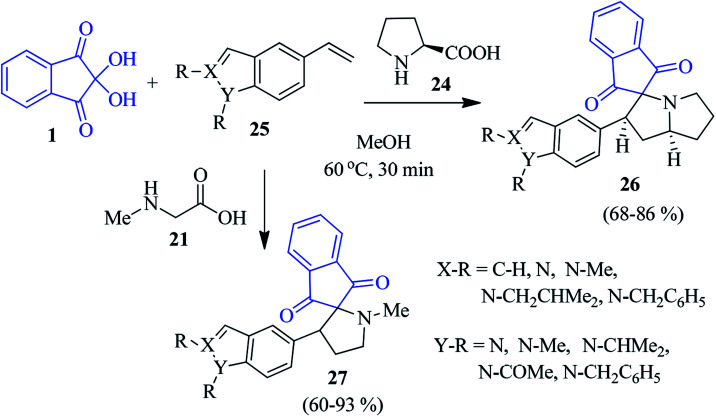
Synthesis of indole/indazole-bearing spiroindenopyrrolidine/pyrrole 26 and 27.

**Scheme 12:**
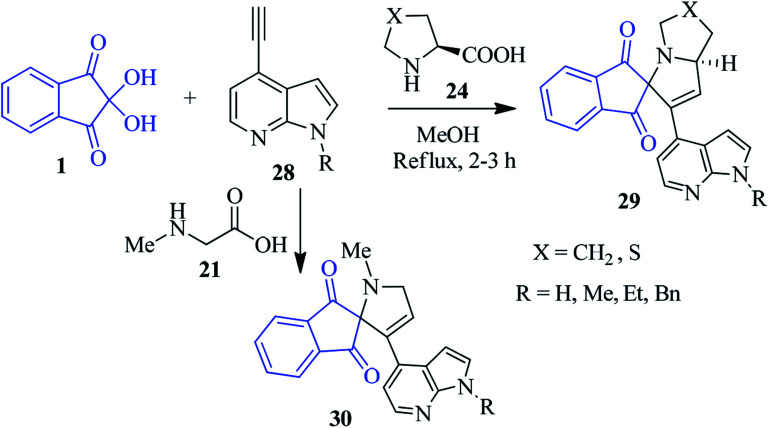
Synthesis of the azaindole-appended spiroindene derivative 29 and 30.

The novel indole/indazole containing spiropyrrolidine compound 26 was prepared by Kamila *et al.* by assembling l-proline 24, ninhydrin 1 and *N*-alkyl vinyl indole/indazole 25 (Scheme 11).^85^ Under similar reaction conditions, sarcosine 21 delivered the corresponding spiropyrrole motif 27 in good yields. The method comprised the 1,3-dipolar cycloaddition reaction between the *in situ* generated azomethine ylide (decarboxylative condensation of ninhydrin and amino acids) and *N*-alkylvinylindole/indazole dipolarophile to obtain the regio- and stereospecific products. Here, a variety of substituted vinyl indoles/indazoles 25 have been engaged to create a library of heterocyclic compounds of biological significance. Later, encouraged by these earlier results, they successfully accessed the azaindole-appended spiro-pyrrolidine skeleton 29/30, employing 1, proline 24 (or sarcosine 21) and *N*-alkyl ethynylazaindole as dipolarophiles 28 (Scheme 12).^86^

**Scheme 13:**
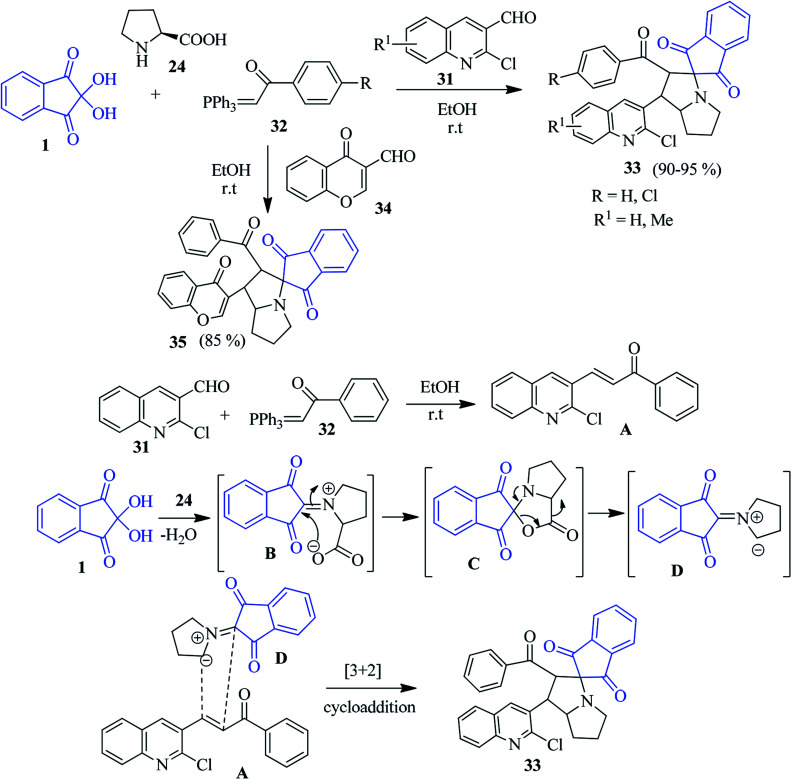
Synthesis of the quinoline/chromene-linked spiro-pyrrolizidine derivatives 33 and 35.

Alizadeh's group outlined a facile and green protocol for the quinolone-based spiro-pyrrolizidine heterocycle 33*via* the one-pot four component sequential combination of ninhydrin 1, l-proline 24, 2-chloroquinoline-3-carbaldehyde 31 and triphenylphosphanylidene 32.^87^ Utilization of chromene-3-carbaldehyde 34 in place of 31 smoothly afforded the corresponding chromene-linked spiro-pyrrolizidine heterocycle 35. The reaction took place with excellent diastereoselectivity. Scheme 13 depicts the mechanism of the formation of the product. Initially, the Wittig reactions of 31 and 32 provided quinolinyl chalcone A, which acts as a dipolarophile. Azomethine ylide D generated from the reaction of ninhydrin 1 and l-proline 24 undergoes a cycloaddition reaction to accomplish the desired product.

**Scheme 14:**
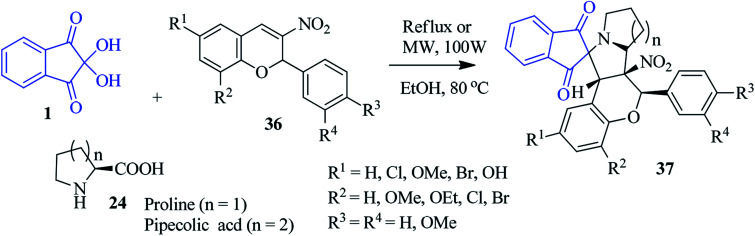
Formation of nitrocoumarin-fused spiro-indanone pyrrolidine derivatives 37.

A fascinating approach to access nitrocoumarin-fused spiro-indanone pyrrolidine compounds 37 was revealed by Nayak and co-workers *via* a three component reaction of 1, l-proline/pipecolic acid 24 and 2-phenyl-nitrochromene dipolarophile 36 (Scheme 14).^88^ The simple method registers the formation of the cycloadducts 37 with excellent regio- and stereospecificity under microwave irradiation as well as conventional heating.

**Scheme 15:**
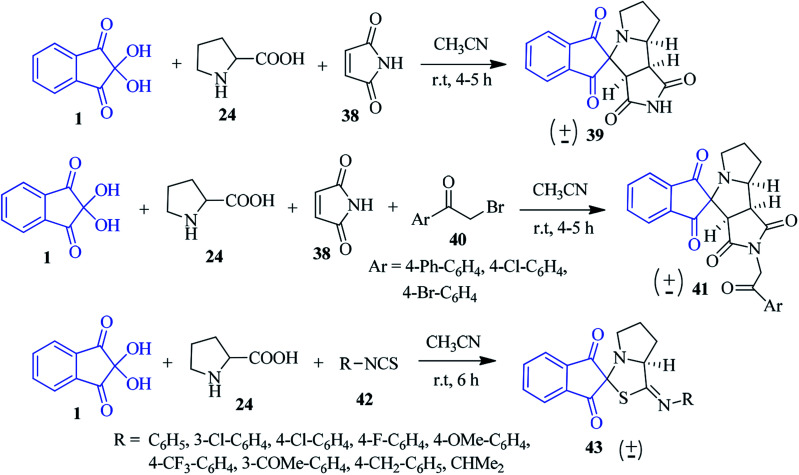
Synthesis of the spiro-indanone pyrrole/pyrrolizine/thiazole derivatives 39, 41 and 43.

Likhar *et al.* synthesized a library of potentially bioactive spiro-indanone pyrrole/pyrrolizine derivatives 39 by using the [3 + 2] dipolar cycloaddition reaction of ninhydrin 1, proline 24 with maleimide 38 (malic anhydride, 2-benzyl-2-methylcyclopent-4-ene-1,3-dione and isothiocyanates also used as dipolarophiles) without any catalyst in CH_3_CN (Scheme 15).^89^ Different α-amino acids, such as thiazolidine-4-carboxylic acid, leucine, valine, phenyl alanine and methionine were employed for constructing the diverse substituted spiro products in good yields. The scope of the reaction could be further extended utilizing phenacyl bromide 40 leading to *N*-substituted analogue 41. Interestingly, various functionalized isothiocyanates 42 were utilized to obtain the corresponding spiro-thiazole derivative 43 under ambient conditions (Scheme 15). The stereochemical assignments of the products were made on the basis of a single crystal X-ray diffraction study.

**Scheme 16:**
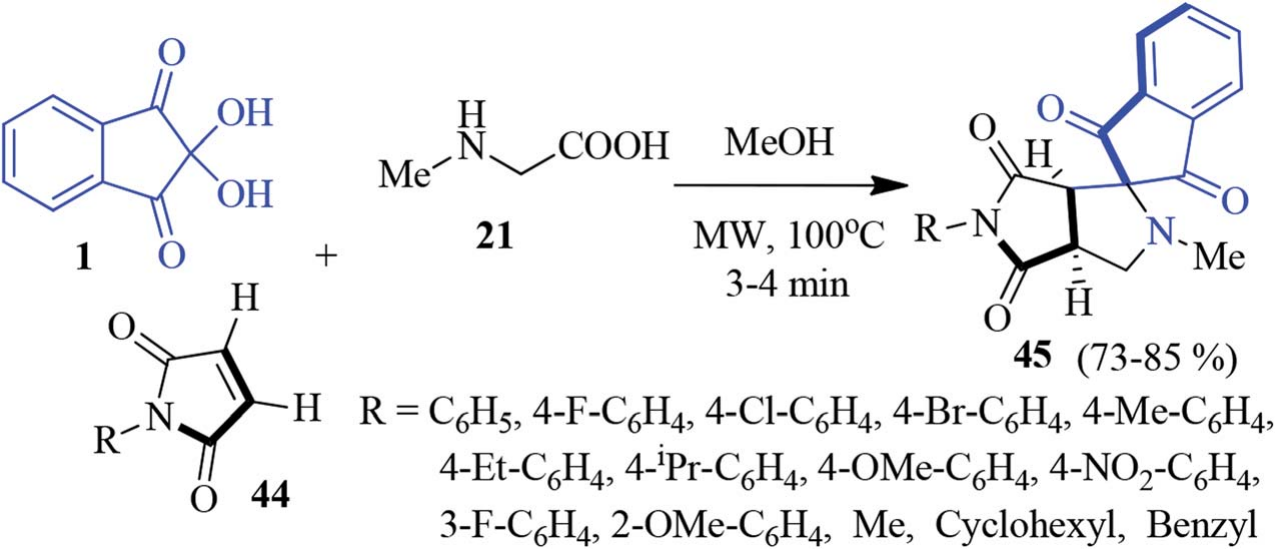
Synthesis of bioactive spiro indeno-pyrrolopyrrole derivatives 45.

A new class of spiro indeno-pyrrolopyrrole derivatives 45 has been accomplished through the microwave-assisted 1,3-dipolar cycloaddition reaction of maleimides 44, ninhydrin 1 and sarcosine 21 (Scheme 16).^90^ A series of maleimides differing in the aryl part with electron releasing and electron withdrawing substituents were successfully incorporated. Importantly, despite the presence of two stereogenic centres in the cycloadduct 45, only one diastereomer has been exclusively obtained. The authors successfully grew single crystals suitable for X-ray analysis for the complete stereochemical assignments. The synthesized compounds were screened for antimycobacterial properties, and AChE inhibition activity, showing promising results.

**Scheme 17:**
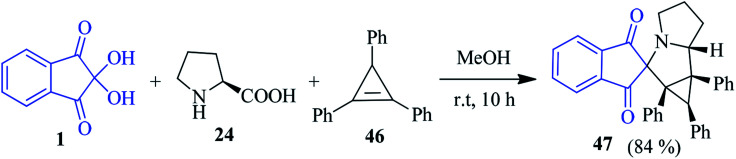
Synthesis of spiro-indanone pyrrolizine-fused cyclopropane system 47.

The construction of a biologically relevant spiro-indanone pyrrolizine-fused cyclopropane system 47 with quaternary stereocentres has been demonstrated by the Stepkov group using the 1,3-dipolar cycloaddition of the stable ninhydrin-derived azomethine ylide to cyclopropenes 46 (Scheme 17).^91^ The [3 + 2] cycloaddition reaction proceeded smoothly, where the highly reactive and unstable cyclopropene was trapped into the reaction process. A DFT computational study was also performed to reveal factors controlling the regio- and stereoselectivity on the observed reactions.

### Cage-like and dispiro compounds

**Scheme 18:**
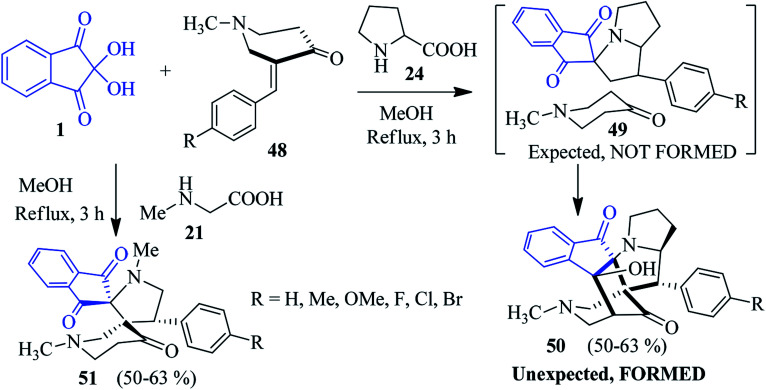
Synthesis of spiro indeno cage-like compounds 50 and dispiro compounds 51.

Kumar *et al.* uncovered a significant way in which ninhydrin 1, proline 24 and (*E*)-3-arylidene-1-methylpiperidin-4-ones 48 were successfully assembled to fabricate the polycyclic spiroindeno cage-like compounds 50 in refluxing MeOH.^92^ The three-component tandem [3 + 2] cycloaddition reactions of azomethine ylide and dipolarophile 48 resulted in the exclusive formation of the unexpected hexacyclic product 50*via* an intramolecular annulation of the expected compound 49. The formation of a cage-like product was confirmed from single crystal X-ray analysis. Under similar reaction conditions, sarcosine 21 produced dispiro compound 51 (Scheme 18).

**Scheme 19:**
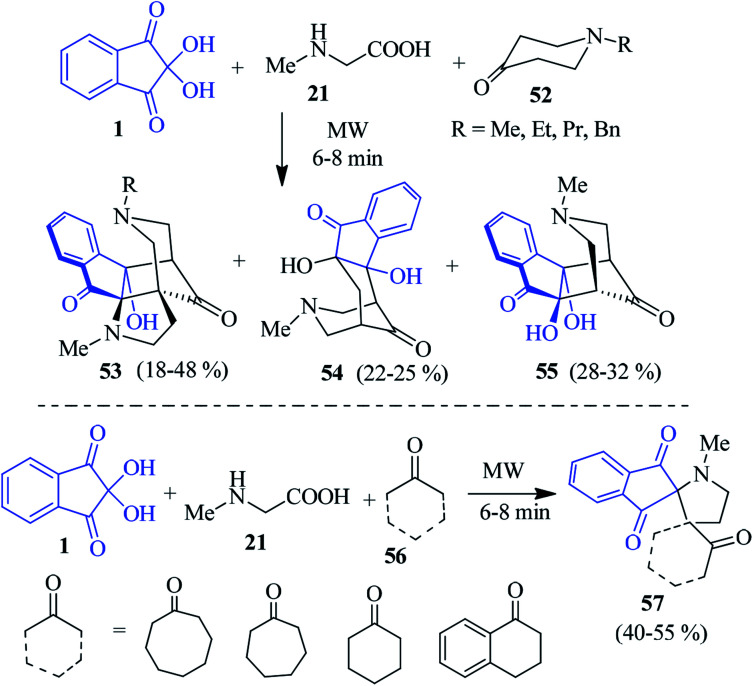
Formation of the cage-like compounds 53 and dispiro heterocycles 57.

Encouraged by the above results, they developed a microwave-assisted solvent-free approach for cage-like compounds and dispiro heterocycles *via* the domino 1,3-dipolar cycloaddition–annulation sequence of reactions.^93^ The reaction of ninhydrin 1 and sarcosine 21 with heterocyclic ketone 52 yielded cage-like compounds 53–55. The heteroatom in the ring might facilitate the annulation of the dispiro compounds initially formed in the reaction, resulting in a cage-like structure. Notably, when the carbocyclic ketones 56 were engaged instead of 52, a new class of dispiro heterocycles 57 was obtained (Scheme 19).

**Scheme 20:**
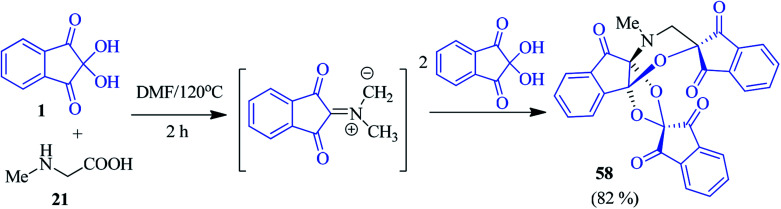
Synthesis of the *N*-methylmorpholine fused dispiroindanone compound 58.

An interesting access to the *N*-methylmorpholine fused dispiroindanone compound 58 was accomplished by Arumugam and co-workers *via* a sequential pseudo four-component cascade cycloaddition reaction involving ninhydrin 1 and sarcosine 21 in DMF solvent (Scheme 20).^94^ The formation of the unusual cycloadduct 58 was supported by DFT calculations, as well as single crystal X-ray diffraction analysis.

**Scheme 21:**
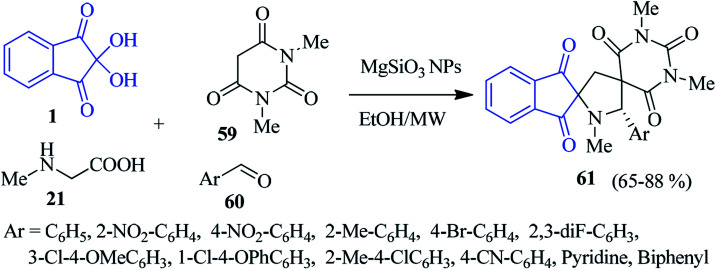
Construction of dispiropyrrolidine scaffold 61.

Efficient magnesium silicate nanoparticles (MgSiO_3_ NPs) catalyzed the multicomponent reaction of ninhydrin 1, sarcosine 21, *N*,*N*-dimethylbarbituric acid 59 and aromatic aldehyde 60 to achieve dispiropyrrolidine derivatives 61, and has been outlined by Koodlur and co-workers (Scheme 21).^95^ The reaction proceeded rapidly, completing within 1–1.5 h under microwave irradiation. The synthesized products were examined for biological evaluation, showing interesting antibacterial activity and antiproliferative activity against tested cell lines.

## 4. Synthesis of spiro-indeno pyrans

**Scheme 22:**
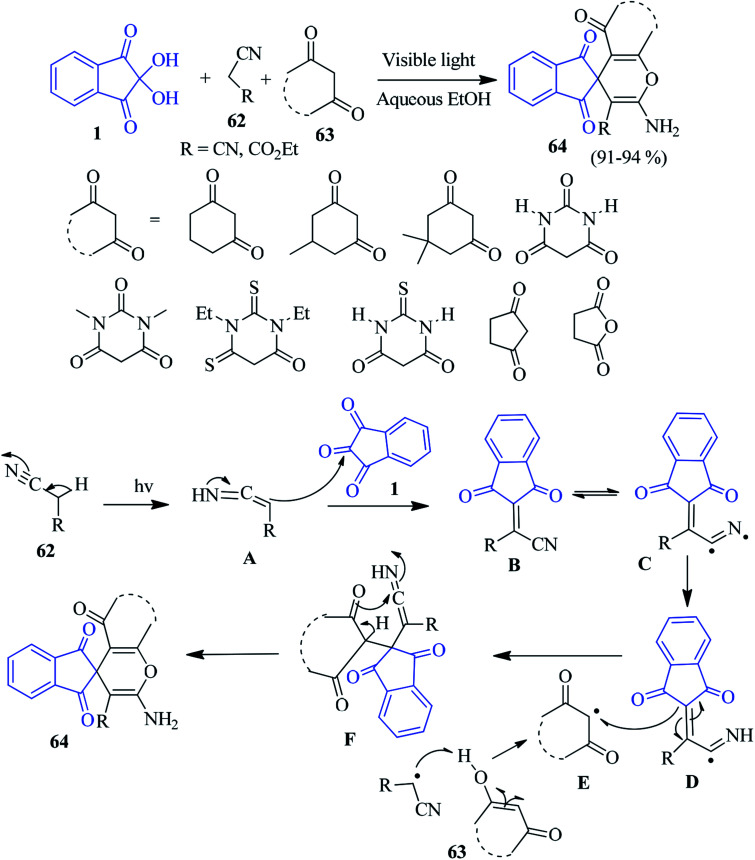
Green synthesis of spiroindeno-pyran derivatives 64.

A convenient and eco-friendly method for the synthesis of spiroindeno-pyran derivatives 64 has been developed by the Siddiqui group *via* a one-pot three component reaction of ninhydrin 1, active methylene compounds 62 and 1,3-dicarbonyl 63, employing a compact fluorescent lamp as a source of light.^96^ The reaction undergoes a smooth transformation of a variety of 1,3-dicarbonyl compounds, resulting in good yields of the corresponding products. A plausible mechanism is offered in Scheme 22. The reaction is initiated by visible light promoting the homolytic fission of the C–H bond of the active methylene compound 62. This homolytic fission and fusion of bonds lead to the formation of intermediate B, which upon addition of the 1,3-dicarbonyl compound, produces the spiropyran derivative 64. This protocol allows for the mild, green and sustainable access to desired heterocycles without any additional catalyst.

Very recently, Singh *et al.* introduced a glucose–water system as a new eco-friendly organocatalyst for the construction of spiropyran/spirochromene analogue employing the aforementioned starting materials.^97^ In another report, the Safari group utilized NiFe_2_O_4_@SiO_2_@melamine magnetic nanoparticles^98^ as a recyclable catalyst to obtain similar compounds.

**Scheme 23:**
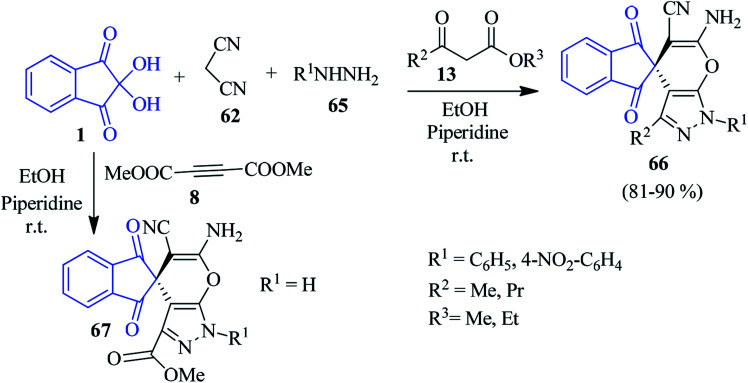
Regioselective synthesis of spiroindeno pyranopyrazole derivatives 66 and 67.

Alizadeh and Bayat elaborated on a one-pot four-component reaction between ninhydrin 1, malononitrile 62, hydrazine derivatives 65 and β-ketoesters 13 to afford spiroindeno pyranopyrazole derivatives 66 regioselectively in EtOH medium in the presence of one drop of piperidine catalyst (Scheme 23).^99^ Employment of dimethylacetylenedicarboxylate (DMAD) 8 in place of 13 resulted in a spiro compound 67.

**Scheme 24:**
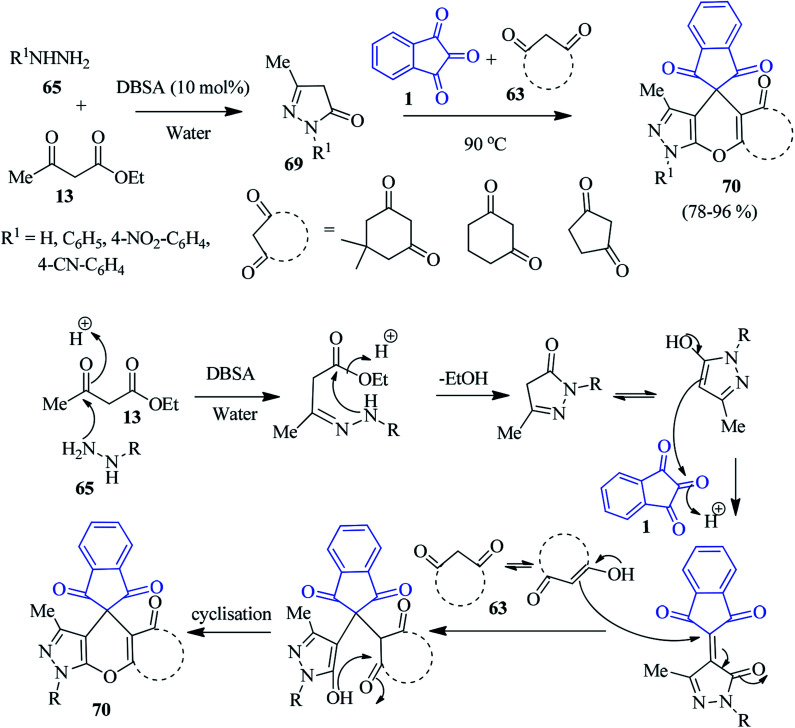
Water-mediated synthesis of the spiroindeno-pyranopyrazole derivatives 70.

Later, Das *et al.* introduced an appealing approach to access the spiro pyranopyrazoles 70, involving dodecylbenzenesulphonic acid (DBSA) as a Brønsted acid–surfactant-combined catalyst in aqueous medium.^100^ The sequential reaction comprises the tandem Knoevenagel/Michael addition reaction followed by the dehydrative cyclisation of pyrazolone derivatives 69 (prepared from ethylacetoacetate 13 and hydrazines 65), cyclic 1,3-diketones 63, and ninhydrin 1 (Scheme 24). The synthetic strategy is operationally simple, economical, and environmentally benign, delivering target compounds in good yields (78–96%).

**Scheme 25:**
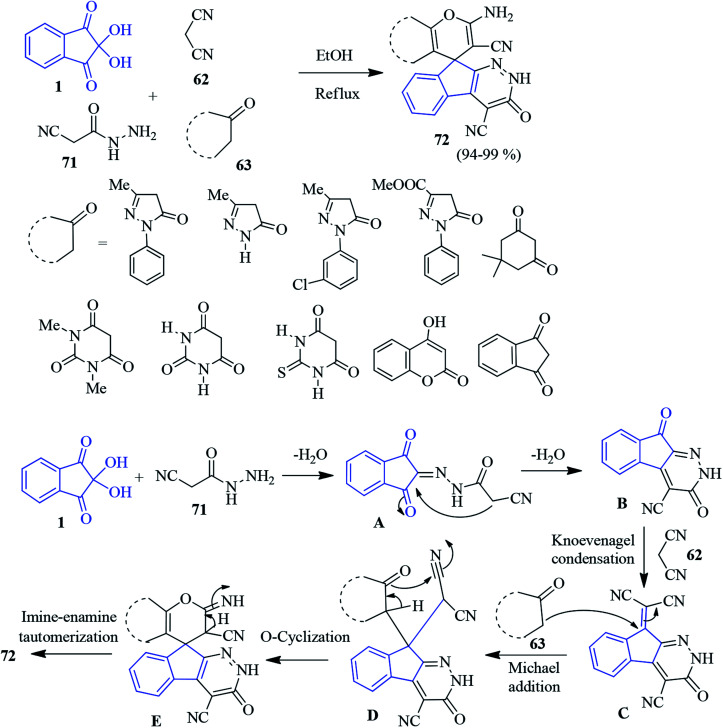
Synthesis of spiroindeno pyranopyridazine derivatives 72.

Recently, Bayat and Hosseini published an efficient one-pot protocol for the synthesis of spiro indeno pyranopyridazine derivatives 72 involving cyanoacetohydrazide 71, ninhydrin 1, malononitrile 62 and different cyclic CH-acids 63 in refluxing EtOH.^101^ According to the mechanism, ninhhydrin 1 and cyanoacetohydrazide 71 condenses to form azomethine intermediate A. Subsequently, the intramolecular cyclisation of A generates indeno-pyridazine intermediate B. The Knoevenagel condensation of malononitrile 62 produces C. Then, the Michael addition of cyclic CH-acids 63 affords intermediate D. Finally, cyclisation followed by imine–enamine tautomerization results stable product 72 (Scheme 25). The products are examined for biological activity.^102^ It has been found that some of the compounds exhibit pronounced antimicrobial (*E. coli* and *S. aureus*), cytotoxic activity (on lung cancer cells, prostate cancer cells, breast cancer cell line *etc.*) and pro-apoptotic effects.

**Scheme 26:**
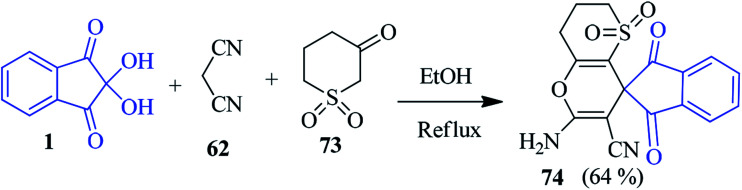
Synthesis of spiroindeno thiopyrano pyran derivative 74.

A new spiro thiopyrano pyran derivative 74 has been prepared by Palchykov and co-workers with the help of dihydro-2*H*-thiopyran-3(4*H*)-one-1,1-dioxide 73, ninhydrin 1 and malononitrile 62 (Scheme 26).^103^ The high reactivity of ketosulfone 73 was exploited to afford the desired product within a short reaction time.

**Scheme 27:**
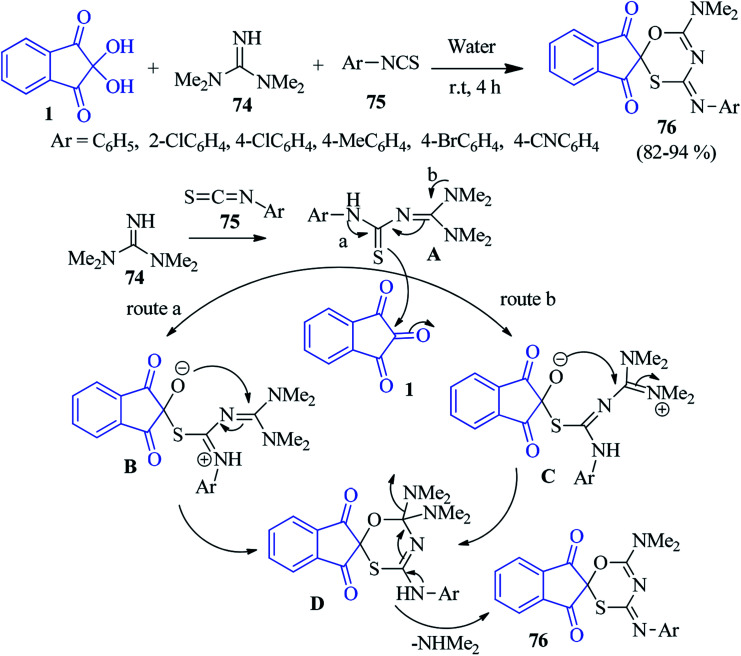
Formation of spiroindeno oxathiazine derivatives 76.

Azizian *et al.* pioneered a concise green method for the synthesis of spiroindeno oxathiazine derivatives 76 by the one-pot three component condensation of tetramethyl guanidine 74, ninhydrin 1 and isothiocyanates 75.^104^ The reaction was fruitful in water at room temperature, where simple filtration afforded novel spiro heterocycle containing oxygen, sulphur and nitrogen. A plausible mechanism is offered in Scheme 27. The nucleophilic attack of tetramethyl guanidine 74 to isothiocyanate 75 produces intermediate A. Subsequently A attacks through its sulphur atom to the central carbonyl of ninhydrin 1 to furnish intermediate B (route a) or C (route b). Finally cyclisation followed by removal of NHMe_2_ leads to the formation of product 76.

## 5. Synthesis of indenoquinoxalines

**Scheme 28:**
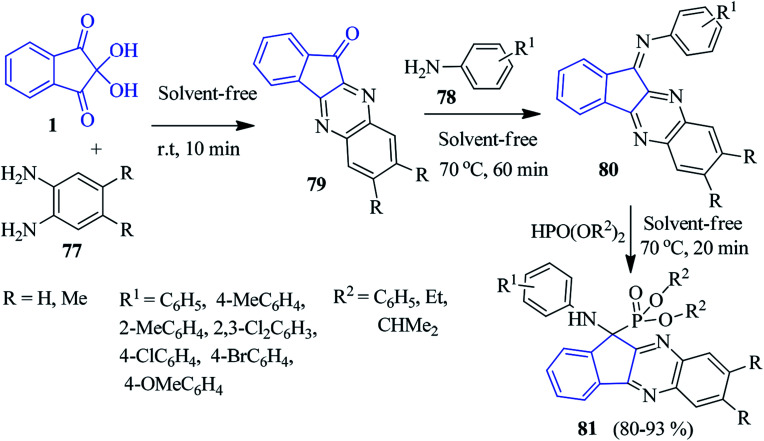
Synthesis of α-aminophosphonate-anchored indenoquinoxalines 81.

In 2015, the Ghahremanzadeh group published a highly efficient protocol for the novel α-aminophosphonate-anchored indenoquinoxaline moiety 81 based on the Kabachnik–Fields reaction involving ninhydrin 1, *o*-phenylenediamine (PDA) 77 and dialkyl or diaryl phosphites 78 (Scheme 28).^105^ The reaction proceeded successfully under solvent-free conditions sequentially with the formation of 79 and 80 without any catalyst, resulting in the desired compound 81 in high yields. It should be mentioned that anilines with an electron donating group reacted smoothly. However, anilines with electron withdrawing groups failed.

**Scheme 29:**
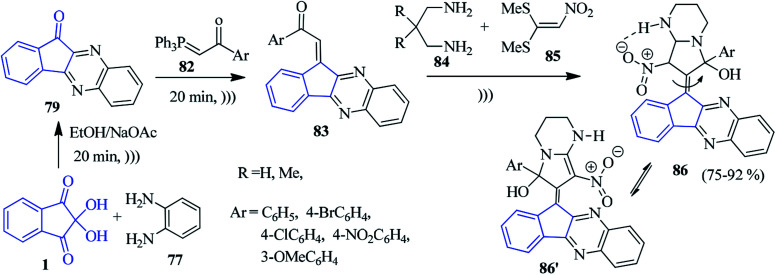
Green synthesis of indenoquinoxalines bearing pyrrolopyrimidine scaffolds 86.

A green regioselective approach to accomplish the production of new indenoquinoxaline compounds containing pyrrolopyrimidine scaffolds 86 was presented by Alizadeh and co-workers (Scheme 29).^106^ The indenoquinoxaline 79 (generated from ninhydrin 1 and PDA 77) was reacted sequentially with 1-aryl-2-(1,1,1-triphenyl-λ^5^-phosphanylidene)ethan-1-one 82 to obtain (*E*)-indenoquinoxaline arylethanone derivatives 83, which were further allowed to react with diamine 84 and 1,1-bis(methylthio)-2-nitromethylene 85 under ultrasound irradiation towards the final compound 86 (which remains in equilibrium with 86′).

## 6. Synthesis of spiro-indenoquinoxaline containing heterocycles

**Scheme 30:**
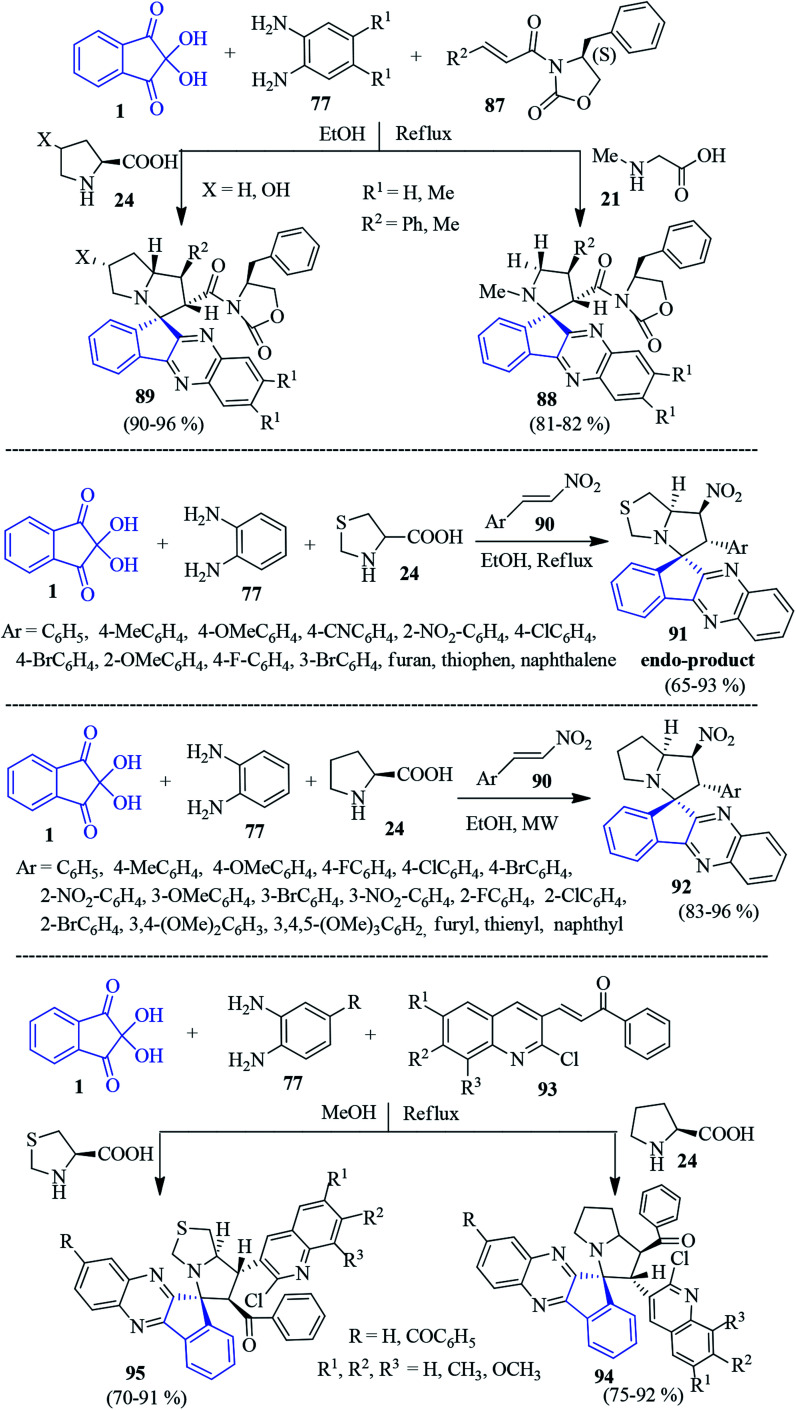
Regioselective synthesis of spiro-indenoquinoxaline derivatives 88, 89, 91, 92, 94, and 95.

In 2015, Jadidi *et al.* demonstrated a one-pot four component reaction of ninhydrin 1, PDAs 77, optically active cinnamoyl-crotonyl oxazolidinone 87 and sarcosine 21/proline 24 to afford the novel chiral spiro-indenoquinoxaline pyrrolidines/pyrrolizidines 88–89 (Scheme 30).^107^ The protocol offers the formation of a complex product (with four contiguous stereogenic centres) from simple starting materials with high regio-, diastereo- (up to 96% dr) and enantioselectivity (up to 99% ee), which proceeded through a 1,3-dipolar cycloaddition reaction of the azomethine ylide in refluxing ethanol.

In the same year, an efficient strategy was introduced by Hamzehloueian for the synthesis of spiro-indenoquinoxaline pyrrolothiazoles 91 involving 1,3-thiazolane-4-carboxylic acid 24, ninhydrin 1, PDA 77 and *trans*-β-nitrostyrene derivatives 90 in refluxing EtOH (Scheme 30).^108^ The reaction proceeded *via* the cycloaddition of *trans*-β-nitrostyrene dipolarophile and *in situ* azomethine ylide generated from 1, 24 and 77. They successfully analysed the mechanism and regioselectivity of the formation of the *endo* product 91 by DFT.

Inspired by the above works, Lakshmi Kantam and Trivedi developed a microwave-assisted protocol towards spiro-indenoquinoxaline pyrrolizine derivatives 92 involving proline 24 (Scheme 30). They successfully evaluated their AChE inhibitory activity.^109^ Rajendran *et al.* disclosed the formation of the quinoline pendant spiro-indenoquinoxaline pyrrolizines 94 involving ninhydrin 1, substituted PDA 77, proline 24 and dipolarophile various quinoline substituted chalcones 93 (Scheme 30).^110^ Immediately after, they synthesized pyrrolothiazole derivatives 95 using thiazolidine-2-carboxylic acid instead of proline. The compounds were screened for *in vitro* antioxidant activities and *in vivo* cytotoxic activity against breast cancer cell line MCF-7 and adenocarcinomic cancer cell line A-549.^111^

**Scheme 31:**
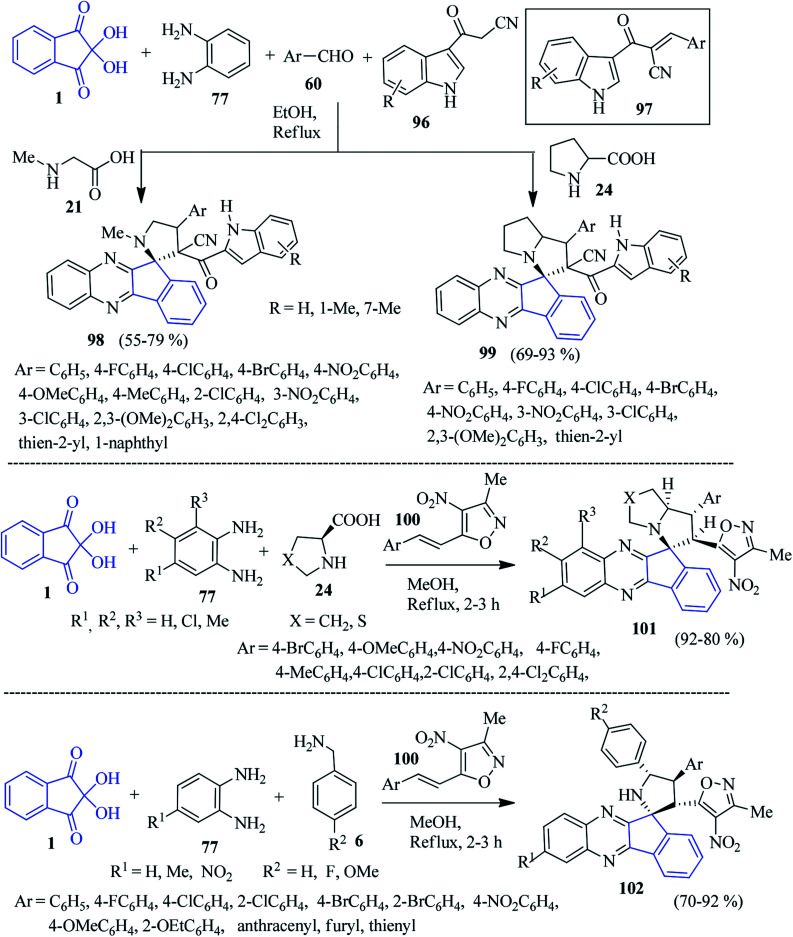
Synthesis of spiro-indenoquinoxaline derivatives 98, 99, 101 and 102.

Novel indole appended spiro-indenoquinoxaline pyrrolidines/pyrrolizidines 98/99 were isolated by Zhu *et al.* through a five-component reaction using ninhydrin 1, PDA 77, amino acids 21/24, 3-cyanoacetyl indoles 96 and aryl aldehydes 60 in EtOH.^112^ The Knoevenagel product 97 generated from the 3-cyanoacetyl indoles 96 and aryl aldehydes 60 acts as dipolarophile (Scheme 31). Notably, the utilization of primary amino acids such as glycine or phenylalanine in this reaction did not afford the target product.

As a part of their synthetic plan, Khurana and Gupta developed a convenient four-component approach to access isoxazole-linked spiro-indenoquinoxaline pyrrolizines 101 involving ninhydrin 1, substituted PDA 77, l-proline/thioproline 24 and 3-methyl-4-nitro-5-styrylisoxazoles 100 in MeOH (Scheme 31).^113^ The catalyst-free simple protocol provides high regioselectivity in a short time frame.

Chowhan and co-workers accomplished a similar type of isoxazole pendant compound 102 through a four-component reaction with high regio- and diastereoselectivity.^114^ They incorporated benzylamine 6, ninhydrin 1, PDA 77 and isoxazole derivatives 100 to achieve the desired product 102*via* the 1,3-dipolar [3 + 2] cycloaddition reaction. In particular, the nature of the substitution and their position on the aromatic rings of styrene (dipolarophile), benzylamines and PDA control the diastereoselectivity of the reaction (Scheme 31). The method is simple, efficient, mild, catalyst-free, column chromatography-free, and does not require any workup procedure.

**Scheme 32:**
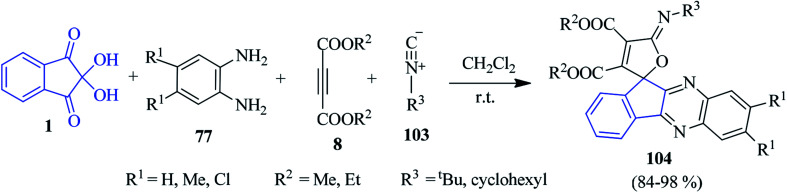
Construction of furan-appended spiro-indenoquinoxaline derivatives 104.

A simple cost-effective method was pioneered by Mahdavinia for the combinatorial synthesis of furan-appended spiro-indenoquinoxaline derivatives 104*via* a one-pot four-component reaction of ninhydrin 1, PDAs 77, DAAD 8 and isocyanides 103 (Scheme 32).^115^ Various substituted benzene-1,2-diamine, methyl and ethyl acetylenedicarboxylates and isocyanides were applied to form the corresponding spiro derivatives in excellent yields. Notably, the reaction did not proceed in protic solvents like EtOH, MeOH, water. However, an excellent yield was obtained in aprotic CH_2_Cl_2_.

### Indenoquinoxaline anchored dispiro scaffolds

**Scheme 33:**
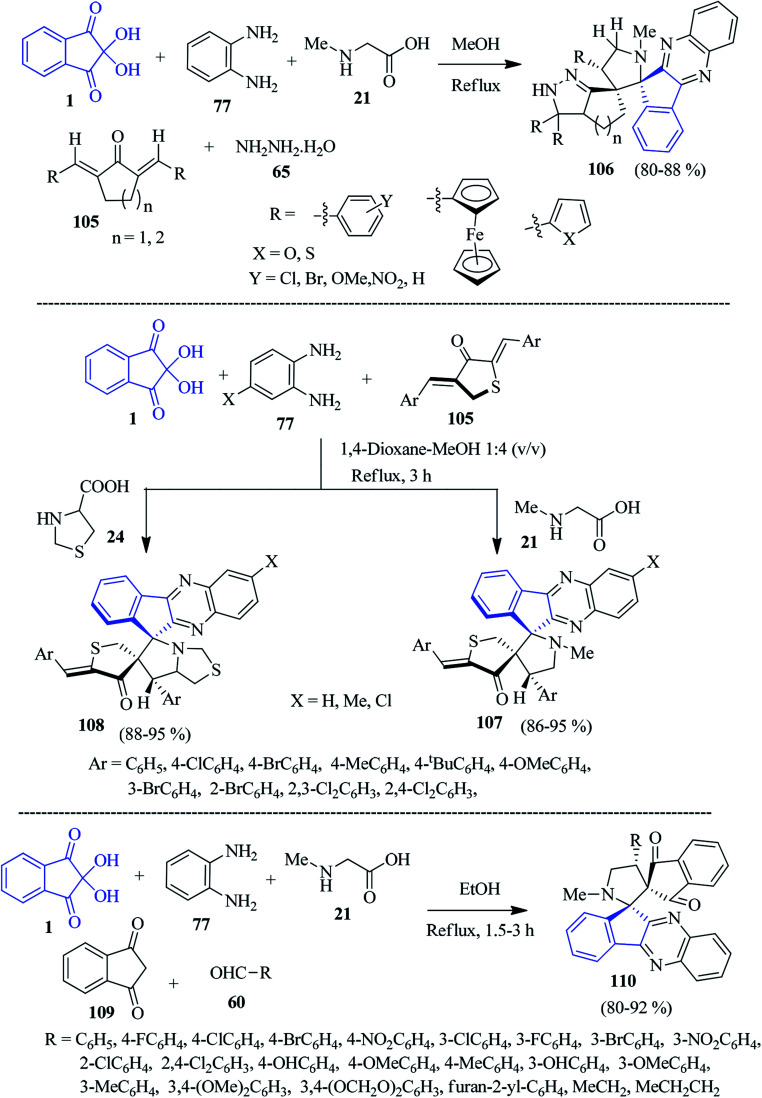
Synthesis of indenoquinoxaline-anchored dispiro scaffolds 106, 107, 108 and 110.

In 2014, Raghunathan and co-workers established a concise route to construct pyrazolo cycloalkane-grafted spiro-indenoquinoxaline pyrrolidines 106 by a sequential five-component reaction involving ninhydrin 1, PDAs 77, sarcosine 21, 2,5-bis-(arylmethylidene)-cycloalkanone 105 and hydrazine hydrate 65*via* the [3 + 2] cycloaddition strategy.^116^ This reaction is applicable to a variety of bis-(arylmethylidene)-cyclopentanone/cyclohexanone systems 105 for the regioselective construction of complex structural entities 106 (Scheme 33).

Later, the Kumar group expanded the scope of the reaction by employing sulphur-containing dipolarophiles, *viz.*, (2*Z*,4*Z*)-2,4-bis-(arylidene)dihydrothiophen-3(2*H*)-ones 105 to build potentially bioactive dihydrothiophenone engrafted spiro-indenoquinoxalines 107 and 108 (Scheme 33).^117^ The reactions are associated with the generation of up to four new contiguous stereocentres, and the formation of two C–C bonds and one C–N bond in a single transformation.

A facile five-component cascade reaction to fabricate novel dispiro-indenoquinoxaline pyrrolidine derivatives 110 was investigated by Li and co-workers utilizing ninhydrin 1, PDA 77, sarcosine 21, 1,3-indanedione 109 and various aldehydes 60.^118^ The reaction took place in high chemo-, regio-, and stereoselective mode. The strategy comprises the cycloaddition of the 1,3-dipole azomethine ylide and dipolarophile simultaneously generated *in situ*, which is complementary to the classical Huisgen synthesis towards the formation of dispiro heterocyclic compounds (Scheme 33).

**Scheme 34:**
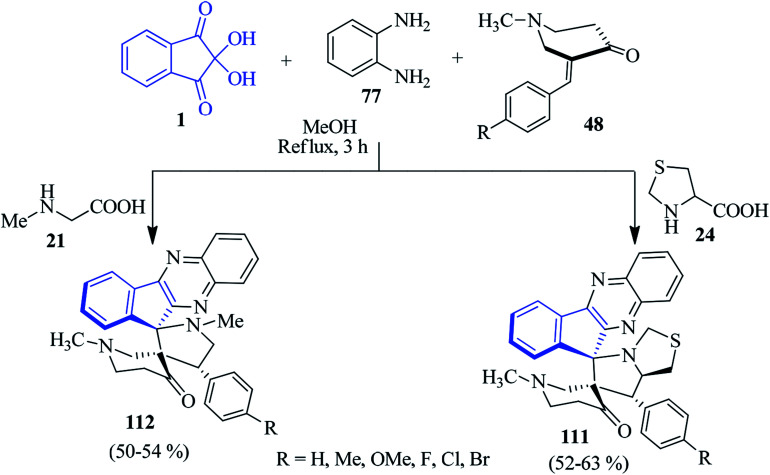
Synthesis of dispiro-indenoquinoxaline hybrid heterocycles 111 and 112.

As part of their studies, the Kumar group exploited ninhydrin 1 and PDA 77 to fabricate dispiro-*N*-methyl-4-piperidone-indenoquinoxaline-pyrrolothiazole/pyrrolidine hybrid heterocycles 111 and 112 by the multicomponent [3 + 2] cycloaddition strategy involving (*E*)-3-arylidene-1-methylpiperidin-4-ones 48 as the dipolarophile (Scheme 34).^92^ These reactions occurred with controlled stereoselectivity, delivering only single isomer.

**Scheme 35:**
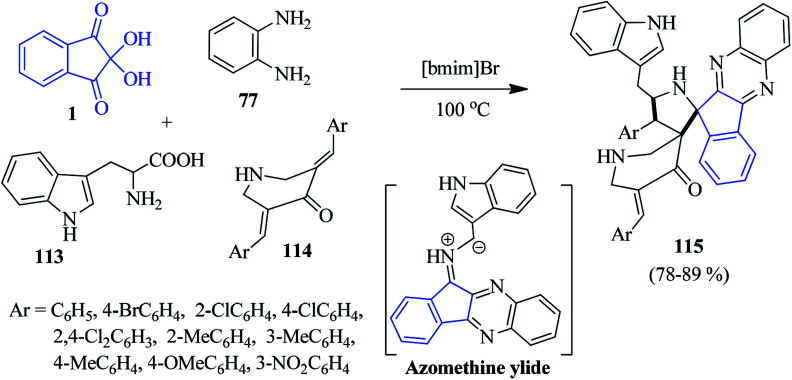
Formation of dispiropyrrolidinyl-piperidone tethered indenoquinoxalines 115.

Very recently, Arumugam and co-workers introduced the ionic liquid [bmim]Br-mediated synthesis of novel dispiropyrrolidinyl-piperidone tethered indenoquinoxaline derivatives 115.^119^ The azomethine ylide generated *in situ* from indenoquinoxalinone and l-tryptophan 113 (*via* decarboxylative condensation) undergoes a 1,3-dipolar cycloaddition reaction with bis-arylidenepiperidone 114, regioselectively furnishing the hybrid heterocycle 115 (Scheme 35). The authors performed tests for biological activity, as well as a docking study. The synthesized compounds were found to exhibit cholinesterase inhibitory activity (AChE and BChE activity).

## 7. Synthesis of propellanes

**Scheme 36:**
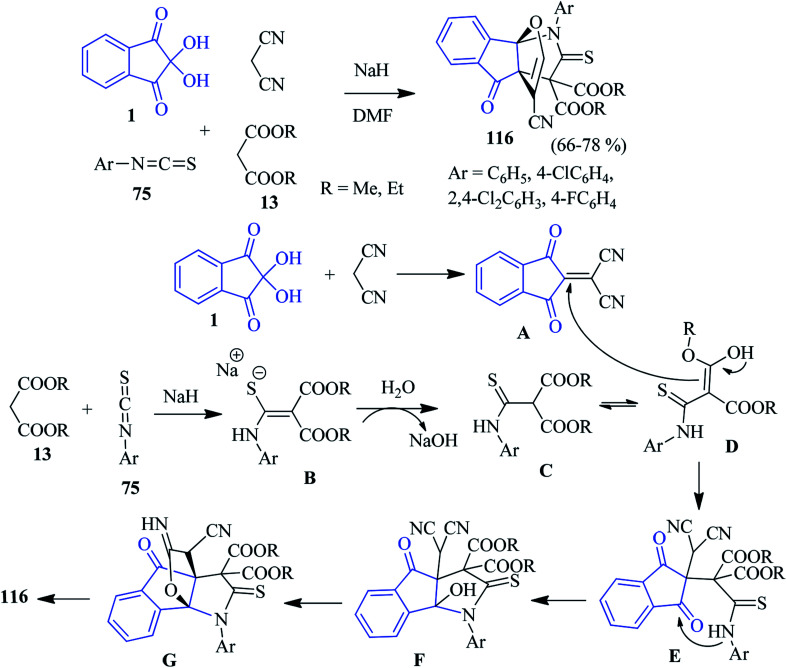
Synthesis of oxa-aza[3,3,3]propellanes 116.

**Scheme 37:**
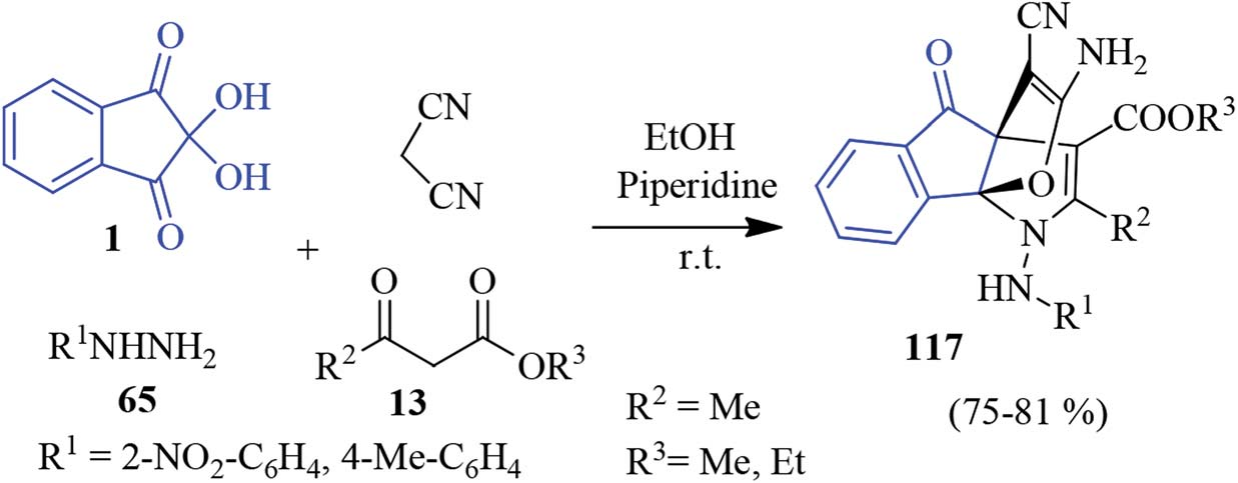
Synthesis of heterocyclic propellanes 117.

In 2014, Alizadeh and co-workers disclosed a sequential four-component approach to accomplish oxa-aza[3,3,3]propellanes 116 by the reaction of the aryl isothiocyanates 75, malonate compounds 13, ninhydrin 1 and malononitrile in the presence of NaH in DMF (Scheme 36).^120^ This methodology offers remarkable chemo- and regioselectivity associated with the formation of five new bonds. The purification of the compounds was carried out without column chromatography. The mechanism of the formation of propellanes 116 is depicted in Scheme 36. The strategy was extended by the authors for the synthesis of a similar type of heterocyclic propellane 117 involving malononitrile, ninhydrin 1, β-ketoesters 13 and hydrazine derivatives 65 in the presence of a piperidine catalyst (Scheme 37).^101^

**Scheme 38:**
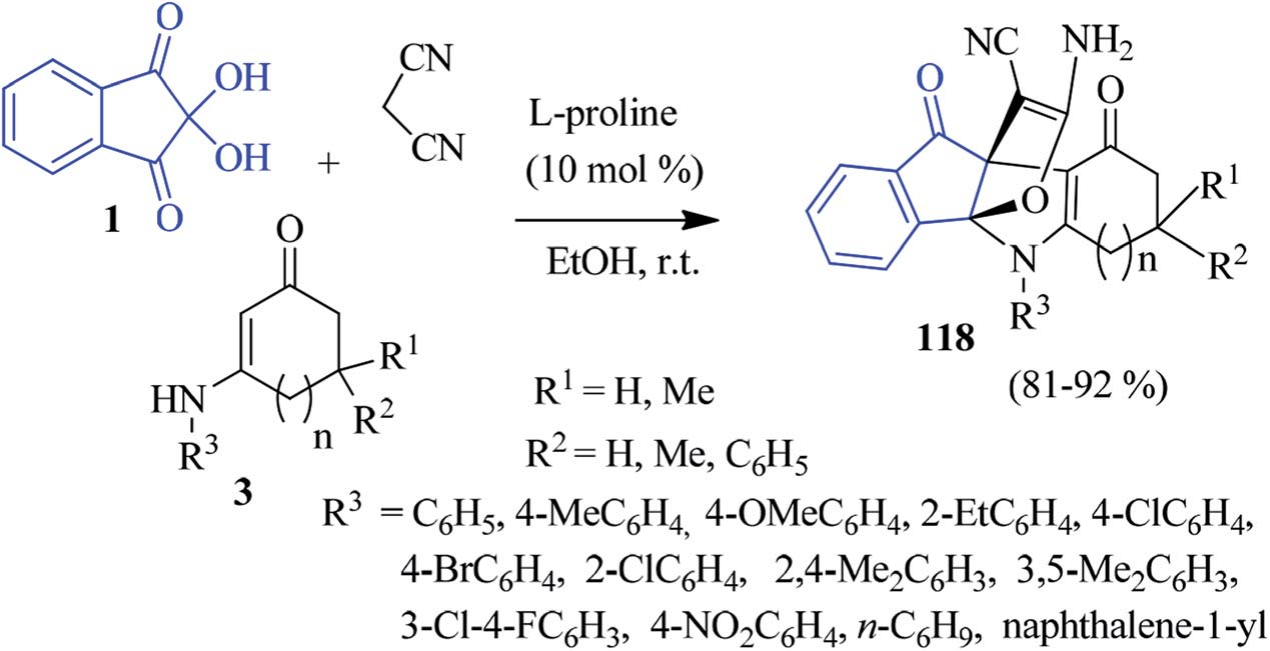
Synthesis of propellanes 118.

An interesting three-component domino reaction of ninhydrin 1, enaminones 3 and malononitrile was reported by Huang *et al.* to access propellanes 118 (Scheme 38).^121^ The reaction was most effective in EtOH in the presence of l-proline as a catalyst (10 mol%) at room temperature. *n*-Butyl, naphthalene-1-yl, and phenyl rings with electron-withdrawing or donating groups on the enaminone ring were well tolerated under the reaction conditions. This reaction comprises the formation of two rings and four bonds by a one-pot procedure.

**Scheme 39:**
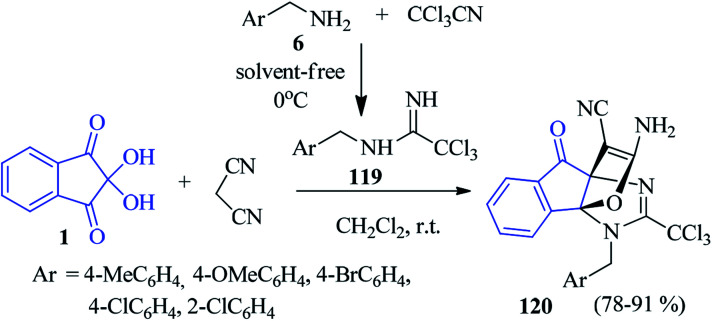
Formation of trichloromethylated [3,3,3]propellanes 120.

Yavari and co-workers found that a tandem reaction of trichloroacetonitrile, substituted benzylamines 6, ninhydrin 1 and malononitrile led to the formation of trichloromethylated [3,3,3]propellanes 120 (Scheme 39). The trichloroacetamidine intermediate 119, generated *in situ* by the addition of trichloroacetonitrile and benzylamines 6 reacted with the Knoevenagel condensation product of ninhydrin 1 and malononitrile to accomplish the desired compound 120.^122^

## 8. Development of diverse molecular scaffolds

In this section, the construction of different ninhydrin-derived skeletons through various rearrangements will be discussed.

### α-Amino acids

**Scheme 40:**
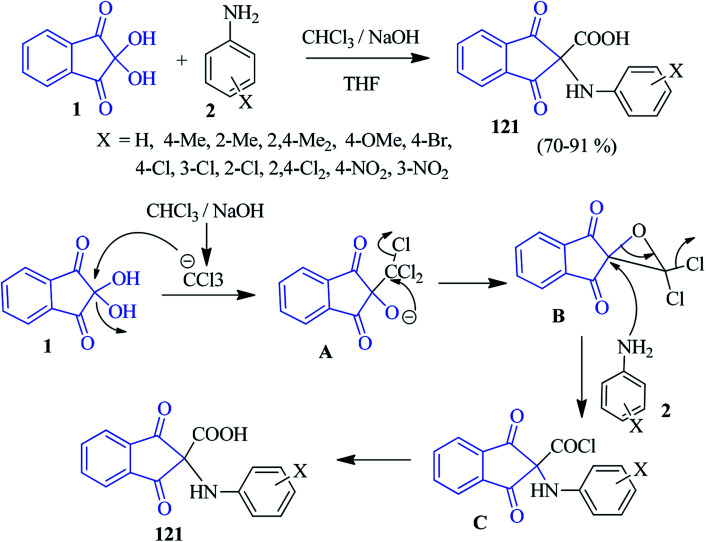
Synthesis of indanone-based α-amino acids 121.

Naeimi and co-workers have developed a facile method to access structurally interesting α-amino acids 121 from ninhydrin 1 and anilines 2 in the presence of CHCl_3_ and NaOH based on the Bargellini reaction in THF medium (Scheme 40).^123^ In this reaction, the ninhydrin core has been exploited as an active carbonyl compound in the Bargellini reaction. Various anilines containing electron donating and withdrawing groups successfully responded under mild conditions. Mechanistically, the NaOH-promoted reaction might proceed *via* deprotonation of CHCl_3_, followed by a nucleophilic attack on ninhydrin and resulting in the dichloro epoxide B. Then, the opening of the epoxide ring by a nucleophilic attack of amine 2 led to the formation of acid chloride C, which after hydrolysis, afforded the desired amino acid 121. Diarylamine compounds also responded well in this transformation. It should be mentioned that other activated carbonyl compounds, such as isatin, acenaphthaquinone and 9,10-phenanthraneraquinone (instead of ninhydrin), did not yield the desired product.

### Pyrrolizines/pyrroles

**Scheme 41:**
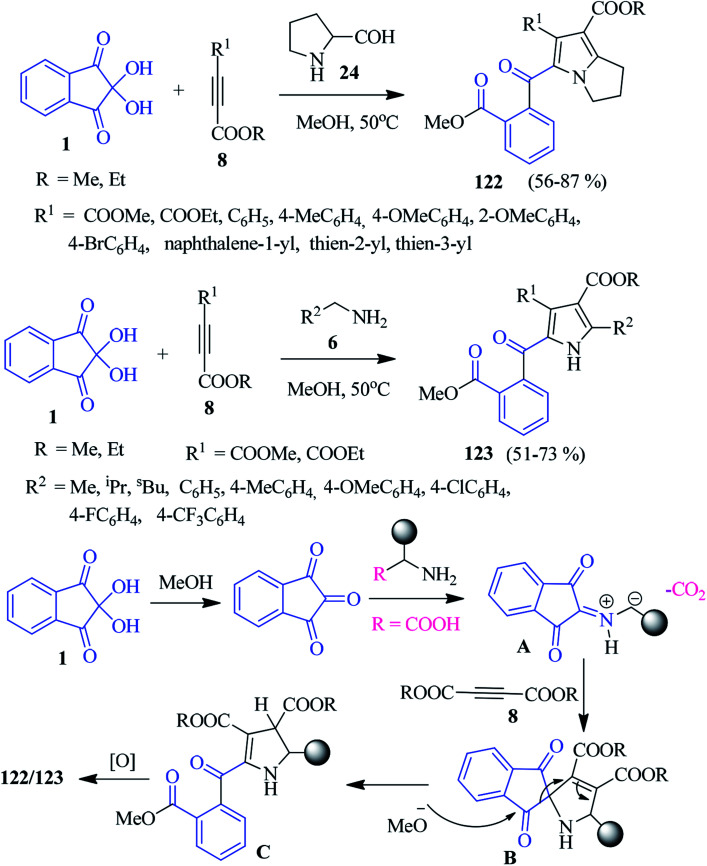
Synthesis of pyrrolizine and pyrrole derivatives 122–123.

A convenient multicomponent methanolysis protocol has been demonstrated by the Meshram group to afford pyrrolizine and pyrrole derivatives 122–123 from ninhydrin 1, alkyne 8 and amines 6/24 (Scheme 41).^124^ The reaction was proposed to go through a [3 + 2] cycloaddition reaction between azomethine ylide and dipolarophile 8. First, ninhydrin 1 transforms into 1,2,3-indanetrione, which reacts with benzyl amine 8 (or amino acids) to obtain the C–N–C dipole intermediate A. Subsequently, the addition of intermediate A to dipolarophile 8 offers spiro-cycloadduct B, which upon methanolysis, leads to the formation of intermediate C. Finally, oxidation affords the desired product 123 (or 122). Notably, the C–C bond of the ninhydrin core is broken here to develop a new skeleton.

### Indolizino-indoles

**Scheme 42:**
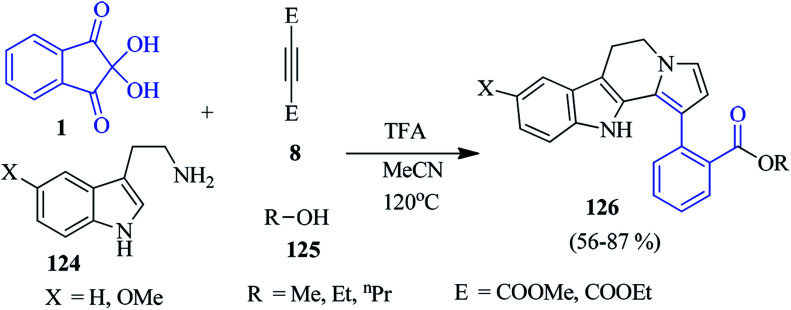
Acid-catalyzed formation of dihydroindolizino[8,7-*b*]indoles 126.

In 2019, Kumbhare *et al.* developed a fascinating four-component approach towards the production of dihydroindolizino[8,7-*b*]indoles 126, engaging ninhydrin 1, substituted tryptamine 124, acetylenic ester 8 and different aliphatic alcohols 125 (Scheme 42).^125^ The reaction proceeded *via* Pictet–Spengler, Michael addition and the nucleophilic addition reaction, leading to the formation of C–C and C–N bonds in the MeCN medium. Notably, the heterocyclic motif was achieved through a double tandem cyclisation in the presence of a CF_3_COOH catalyst.

### Isoquinolinones

**Scheme 43:**
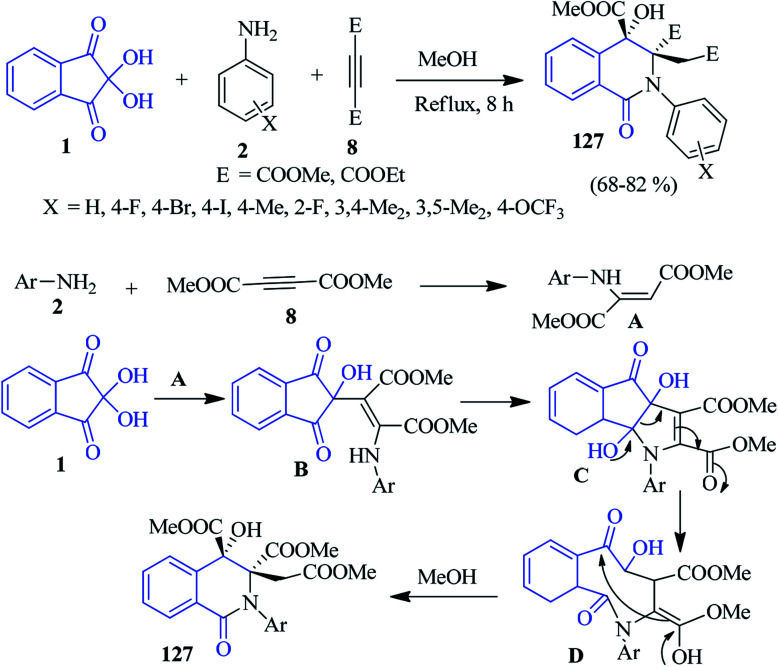
Base-prompted synthesis of dihydroisoquinolin-2-(1*H*)-ones 127.

In the same year, they invented a base-promoted three-component diastereoselective reaction of ninhydrin 1, anilines 2 and acetylenic esters 8 to accomplish *N*-aryl-substituted dihydroisoquinolin-2-(1*H*)-ones 127 in MeOH (Scheme 43).^126^ Initially, the addition of amine 2 and acetylenic esters 8 gives intermediate A, which is subsequently reacted with ninhydrin 1 to form intermediate B. The intramolecular cyclization of B affords intermediate C, which then undergoes a pinacol–pinacolone rearrangement to afford intermediate D. Finally, methanolysis (intramolecular cyclization) offers the desired product 127 with excellent diastereoselectivity. In this reaction, the insertion of nitrogen occurs to form an isoquinolinone scaffold. The relative stereochemistry of the product was confirmed by single crystal X-ray diffraction studies.

### Pyrido-isoquinolinones

**Scheme 44:**
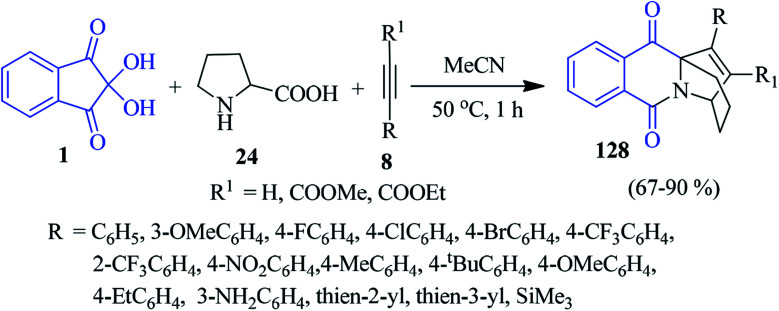
Synthesis of pyrido[1,2-*b*] isoquinoline derivatives 128.

Likhar and co-workers devised a convenient one-pot tandem approach to obtain a library of pyrido[1,2-*b*] isoquinoline derivatives 128, employing readily available ninhydrin 1, proline 24 and alkynes 8 under ambient condition (Scheme 44).^127^ A wide range of aromatic alkynes bearing electron donating and electron withdrawing groups at different positions on the aromatic ring smoothly underwent the reaction to furnish the desired product. This method comprises a [3 + 2] cycloaddition reaction between alkynes and isoquinolium ylide (1,3 dipole) generated *in situ* from ninhydrin and proline. Importantly, two new C–N bonds, three C–C bonds and three new rings are formed in a single step.

### Isoquinolino-quinazoline

**Scheme 45:**
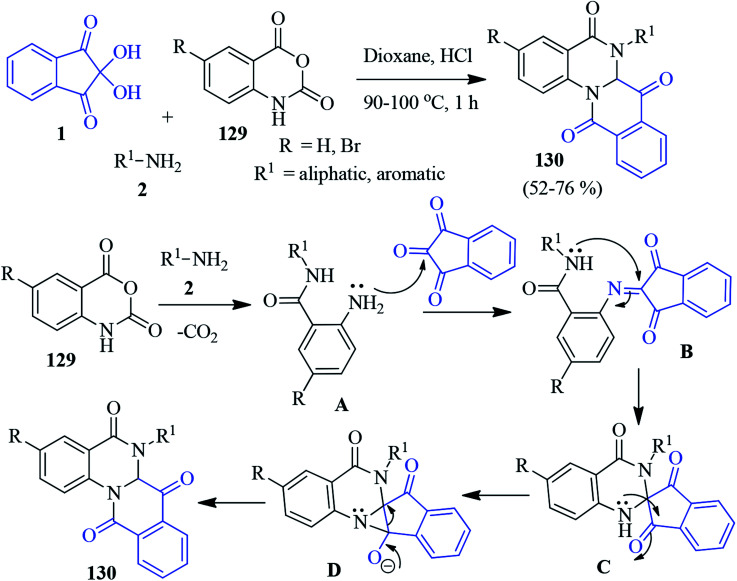
Construction of substituted isoquinolino-quinazolines 130.

A simple and efficient approach for the construction of substituted isoquinolino-quinazoline derivatives 130 has been introduced by the Raghunadh group through a multicomponent reaction employing ninhydrin 1, aliphatic/aromatic amines 2 and isatoic anhydride 129.^128^ The reaction was most effective in 10% HCl in 1,4-dioxane. A plausible mechanism of the tandem cyclization is offered in Scheme 45. Initially, a nucleophilic attack by the primary amine on the carbonyl group of isatoic anhydride followed by decarboxylation leads to compound A, which condenses with the central carbonyl of ninhydrin to give intermediate B. Next, the intramolecular cyclization produces spiro intermediate C, and the nucleophilic attack of the amine on the keto group generates the aziridine intermediate D. Finally, a rearrangement furnishes the tetracyclic quinazolinone derivatives 130.

### Isochromeno-pyrrole

**Scheme 46:**
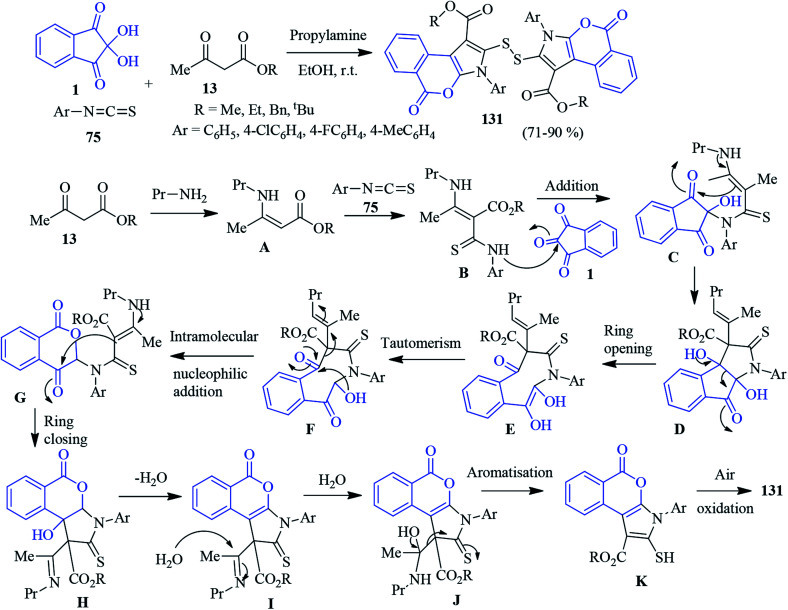
Synthesis of disulfide-linked isochromeno-pyrrole derivatives 131.

A highly convergent one-pot domino protocol was developed by Alizadeh to afford isochromeno-pyrroles containing a disulfide linkage 131 (Scheme 46).^129^ The sequential reaction involves the assembly of enamines (generated from a β-keto ester 13 and propylamine) with aryl isothiocyanates 75, producing an intermediate that is trapped by ninhydrin 1 to deliver the desired compound. The formation of the isochromeno-pyrrole skeleton was confirmed by X-ray crystal structure. A plausible pathway for the formation of the product is depicted in Scheme 46. The nucleophilic addition of enamine A (produced from propylamine and β-ketoester 13) to the electrophilic centre of the aryl isothiocyanate 75 takes place to generate intermediate B. Then, intermediate B attacks the central carbonyl of ninhydrin (B to C) followed by an intramolecular azaene reaction to furnish intermediate D. The eight-membered lactam intermediate E (produced *via* ring opening) then undergoes tautomerisation, resulting in the keto form F. Nucleophilic attack by hydroxyl group affords intermediate G, which after ring closing, gives intermediate H. Dehydration (H to I) followed by aromatisation produces the isochromeno-pyrrole skeleton K. Finally, air oxidation leads to the formation of the desired product 131.

### Benzofuran

**Scheme 47:**
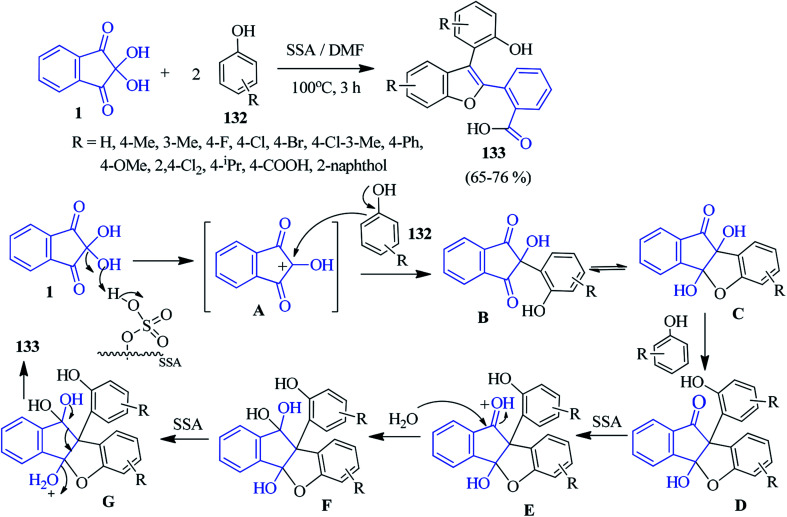
Acid-catalyzed formation of multi-functionalized benzofuran derivatives 133.

Pramanik and Kundu described an efficient method for the construction of biologically relevant multi-functionalized benzofurans 133 involving ninhydrin 1 and substituted phenols 132 in a 1 : 2 molar ratio. In this reaction, the environmentally benign silica sulphuric acid (SSA) was used as a heterogeneous acid catalyst to carry out the rearrangement in DMF medium.^130^ The salient features of this work are its operational simplicity, cost-effectiveness, metal-free property, good yield and use of recyclable SSA. A possible reaction mechanism has been proposed in Scheme 47. The X-ray data unambiguously supports the formation of substituted benzofuran derivatives 133.

### Chromeno-isoindolo-pyrrole

**Scheme 48:**
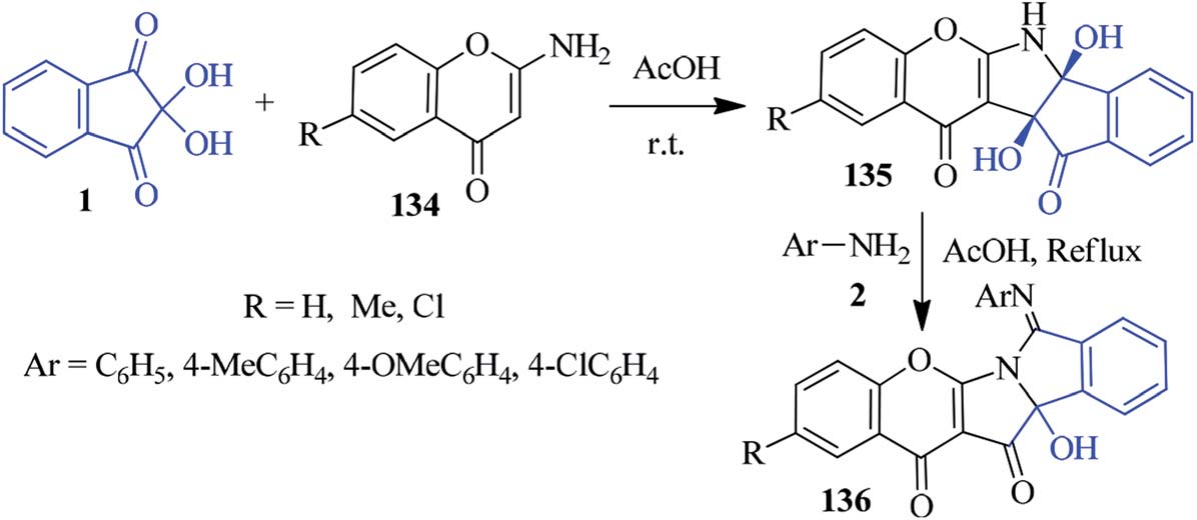
Acid-catalyzed synthesis of chromeno[2,3-*b*]isoindolo[1,2-*e*]pyrroles 136.

Bandyopadhyay *et al.* described a sequential one-pot protocol to access the chromeno[2,3-*b*]isoindolo[1,2-*e*]pyrrole scaffold 136*via* acid-catalyzed rearrangement.^131^ At room temperature, the stirring of ninhydrin 1 and 2-aminochromen-4-ones 134 in AcOH furnished chromeno-indeno-pyrrole derivatives 135, which upon reflux with aromatic amines 2 in AcOH, delivered the final product 136 (Scheme 48).

### Isochroman-1,4-diones

**Scheme 49:**
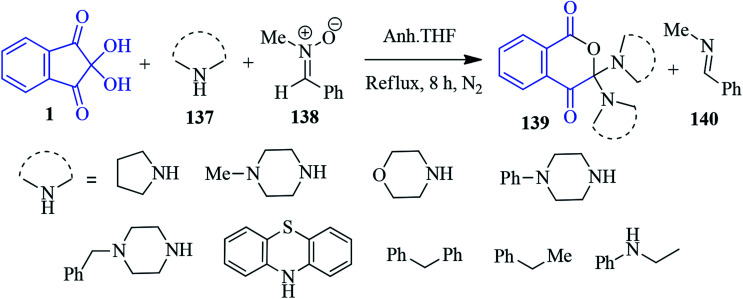
Synthesis of 3,3-disubstituted isochroman-1,4-diones 139.

The synthesis of 3,3-disubstituted isochroman-1,4-dione 139 was reported by Deepthi *et al.* involving ninhydrin 1, secondary amines 137 and *N*-methyl-*C*-phenyl nitrone 138 (Scheme 49).^132^ In fact, nitrone acts as an oxygen atom donor to afford desired product 139 and imine 140 as side product. Interestingly, most of the isochroman-1,4-dione derivatives were found to be fluorescent in solution with high quantum yields.

### Spirofuran-isoindole

**Scheme 50:**
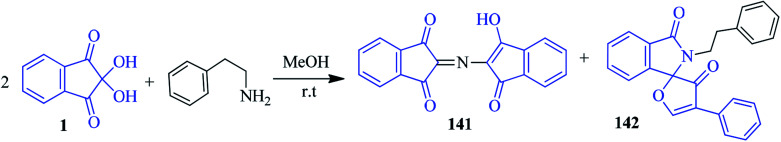
Formation of benzo-fused spiroheterocyclic compound 142.

We complete our discussion by considering an interesting example of a ninhydrin reaction with amine. The reaction of ninhydrin with amines usually leads to a single product known as Ruhemann's purple. However, the Quevedo group disclosed that the reaction between ninhydrin 1 and phenylethylamine (2 : 1 ratio) generated Ruhemann's purple 141, along with the formation of the new benzo-fused spiroheterocyclic system 142 (Scheme 50).^133^ The spiro compound 142 was formed through the rearrangement of the ninhydrin ring structure. The structure of the newly formed compound was established using correlation spectroscopy and single crystal X-ray diffraction study.

## 9. Conclusions

Being a unique tricarbonyl compound, ninhydrin has already left its imprint in organic chemistry, biochemistry, analytical chemistry and the forensic sciences. It has been successfully employed as a potential synthon by exploiting its most electrophilic C-2 position to react with various nucleophiles. In this review, we surveyed progress from the last six years on the development of diverse molecular scaffolds employing ninhydrin in multicomponent reactions. These reactions led to the construction of complex molecular systems, such as indeno-fused heterocycles, spiro-indeno heterocycles, quinoxalines, propellanes, cage-like compounds and dispiro heterocycles. In addition, the formation of several novel molecular architectures *via* different rearrangement was highlighted. Some of the rearrangements involved the breaking of the ninhydrin core to achieve various heterocyclic skeletons. Interesting examples of the regio- and stereoselective synthesis of biologically relevant compounds have also been disclosed. We believe that this review will attract the attention of researchers in the field of chemistry and biology.

## Conflicts of interest

The author declares no conflicts of interest.

## Supplementary Material
